# Transcriptome and small RNAome profiling uncovers how a recombinant begomovirus evades RDRγ-mediated silencing of viral genes and outcompetes its parental virus in mixed infection

**DOI:** 10.1371/journal.ppat.1011941

**Published:** 2024-01-12

**Authors:** Margaux Jammes, Victor Golyaev, Alejandro Fuentes, Nathalie Laboureau, Cica Urbino, Clemence Plissonneau, Michel Peterschmitt, Mikhail M. Pooggin

**Affiliations:** 1 PHIM Plant Health Institute, University Montpellier, CIRAD, INRAE, IRD, Institute Agro, Montpellier, France; 2 Center for Genetic Engineering and Biotechnology, Habana, Cuba; 3 GAUTIER Semences, Route d’Avignon, Eyragues, France; The Ohio State University, UNITED STATES

## Abstract

Tomato yellow leaf curl virus (TYLCV, genus *Begomovirus*, family *Geminiviridae*) causes severe disease of cultivated tomatoes. Geminiviruses replicate circular single-stranded genomic DNA via rolling-circle and recombination-dependent mechanisms, frequently generating recombinants in mixed infections. Circular double-stranded intermediates of replication also serve as templates for Pol II bidirectional transcription. IS76, a recombinant derivative of TYLCV with a short sequence in the bidirectional promoter/origin-of-replication region acquired from a related begomovirus, outcompetes TYLCV in mixed infection and breaks disease resistance in tomato *Ty-1* cultivars. *Ty-1* encodes a γ-clade RNA-dependent RNA polymerase (RDRγ) implicated in Dicer-like (DCL)-mediated biogenesis of small interfering (si)RNAs directing gene silencing. Here, we profiled transcriptome and small RNAome of *Ty-1* resistant and control susceptible plants infected with TYLCV, IS76 or their combination at early and late infection stages. We found that RDRγ boosts production rates of 21, 22 and 24 nt siRNAs from entire genomes of both viruses and modulates DCL activities in favour of 22 and 24 nt siRNAs. Compared to parental TYLCV, IS76 undergoes faster transition to the infection stage favouring rightward transcription of silencing suppressor and coat protein genes, thereby evading RDRγ activity and facilitating its DNA accumulation in both single and mixed infections. In coinfected *Ty-1* plants, IS76 efficiently competes for host replication and transcription machineries, thereby impairing TYLCV replication and transcription and forcing its elimination associated with further increased siRNA production. RDRγ is constitutively overexpressed in *Ty-1* plants, which correlates with begomovirus resistance, while siRNA-generating DCLs (DCL2b/d, DCL3, DCL4) and genes implicated in siRNA amplification (α-clade RDR1) and function (Argonaute2) are upregulated to similar levels in TYLCV- and IS76-infected susceptible plants. Collectively, IS76 recombination facilitates replication and promotes expression of silencing suppressor and coat proteins, which allows the recombinant virus to evade the negative impact of RDRγ-boosted production of viral siRNAs directing transcriptional and posttranscriptional silencing.

## Author summary

In plants, endogenous and antiviral RNAi mediated by Dicers generating siRNAs and Argonautes binding siRNAs to silence plant and viral genes transcriptionally and/or posttranscriptionally can be amplified by RNA-dependent RNA polymerases (RDRs) of α-clade generating precursors of secondary siRNAs. To establish successful infection, viruses evade or suppress antiviral RNAi. Here we undertook small RNAome and transcriptome profiling to uncover how a recombinant ssDNA begomovirus evades repressive siRNAs, overcomes resistance in *Ty-1* tomato cultivars mediated by a γ-clade RDR and outcompetes parental viruses in mixed infection. We found that the recombination event within the intergenic region carrying bidirectional promoter and origin-of-replication elements facilitates viral DNA replication and promotes rightward transcription of RNAi suppressor and coat protein genes. This allows the recombinant virus to evade the negative impact of RDRγ-boosted production of 22 and 24 nt siRNAs which effectively repress the parental virus, leading to its elimination in mixed infection.

## Introduction

Tomato yellow leaf curl disease (TYLCD) is caused by several single-stranded (ss)DNA viruses belonging to the genus *Begomovirus* of the family *Geminiviridae* (geminiviruses). TYLCD is one of the major threats for tomato cultivation worldwide due to severe leaf symptoms, plant stunting and flower abortion, resulting in reduced tomato yield. In the Mediterranean basin, the disease is mainly caused by tomato yellow leaf curl virus (TYLCV), tomato yellow leaf curl Sardinia virus (TYLCSV) and their recombinants.

Geminiviruses replicate in the plant cell nucleus via rolling-circle and recombination-dependent mechanisms and encapsidate circular ssDNA products of rolling-circle replication into geminate (twinned icosahedra) virions [1,2]. Circular double-stranded (ds)DNA intermediates of both replication mechanisms also serve as templates for Pol II-mediated transcription of viral genes. As typical monopartite begomoviruses, TYLCV and TYLCSV possess six genes transcribed by Pol II bidirectionally from the virion and complementary strands of circular dsDNA of ~2.8 Kbp. The rightward (virion strand) genes V1 and V2 encode the coat protein (CP/V1) [3,4] and the strong silencing suppressor (V2) also implicated in movement [5–11]. The leftward (complementary strand) genes (C1-to-C4) encode the replication initiator protein (Rep/C1) [12], the transcriptional activator and silencing suppressor (TrAP/C2) [13,14], the replication enhancer (REn/C3) [15] and the silencing suppressor (C4) also implicated in movement and pathogenicity [5,6,16–18]. The intergenic region contains the origin of replication and, as shown for both monopartite and bipartite begomoviruses, the bidirectional promoter driving Pol II transcription of respectively the C1-C4 mRNA from which Rep/C1 and C4 are translated and the V2-V1 mRNA from which V2 and CP are translated; an additional monodirectional promoter drives Pol II transcription of the C2-C3 mRNA from which TrAP/C2 and REn/C3 proteins are translated and which is 3’-coterminal with the C1-C4 mRNA [19–21]. Several line of evidence indicate that the leftward transcription dominates at early stages of cell infection favouring rolling-circle replication of viral DNA, while the rightward transcription is activated at the later stages favouring encapsidation of viral DNA. As shown for bipartite begomoviruses, the rightward transcription is activated by a concert action of the viral Rep that represses the leftward transcription of its own mRNA [21,22] and the viral TrAP that transactivates the rightward transcription of CP mRNA [21,23], thereby leading to overexpression of the viral CP that encapsidates viral circular ssDNA.

Geminiviruses are transmitted by phloem-feeding insect vectors such as whiteflies, aphids and leafhoppers in a persistent circulative manner. TYLCV and other begomoviruses are transmitted exclusively by the whitefly *Bemisia tabaci*. Due to its small size, high biotic potential, large host range and propensity to develop insecticide resistance, *B*. *tabaci* is a very difficult pest to control. That is why breeding for plant resistance has so far been the most effective strategy to prevent and control begomoviral diseases. In the case of TYLCD, six resistance genes (*Ty-1* to *Ty-6*) available from the wild *Solanum* species have been introgressed into the genome of cultivated tomatoes (*Solanum lycopersicum*) and the *Ty-1* resistant plants are the most cultivated [24–26].

The tomato *Ty-1* gene codes for an RNA-dependent RNA polymerase from the γ-clade (RDRγ) [27]. This gene is similar to the RDRγ genes of the model plant *Arabidopsis thaliana* (RDR3, RDR4 and RDR5) for which no function have been demonstrated yet. The α-clade RDRs of *A*. *thaliana* (RDR1, RDR2 and RDR6) are involved in RNA interference (RNAi), an evolutionarily conserved mechanism that regulates gene expression and defends against invasive nucleic acids such as transposons, transgenes and viruses in most eukaryotes. RNAi is directed by small interfering (si)RNAs which are produced by Dicer or Dicer-like (DCL) family proteins from double-stranded (ds)RNA precursors and get associated with Argonaute (AGO) family proteins forming RNA-induced silencing complexes. In plants, the dsRNA precursors of siRNAs are generated by sense and antisense transcription or transcription of inverted repeats as well as by the activity of RDR1, RDR2 or RDR6 synthesizing complementary strands on their specific single-stranded (ss)RNA templates [28]. DCLs process their respective dsRNA substrates into 21 nt (DCL4), 22 nt (DCL2) and 24 nt (DCL3) siRNAs which are then sorted by AGOs based mostly on their size and 5’-terminal nucleotide identity [29]. In *A*. *thaliana* infected with a bipartite begomovirus, DCL4, DCL2 and DCL3 produce respectively 21, 22 and 24 nt viral siRNAs covering both strands of the entire virus genome [30,31]. The majority of begomoviral siRNAs are generated independently of the activities of RDR1, RDR2 or RDR6 or the plant-specific DNA-dependent RNA polymerases Pol IV and Pol V [30,31], suggesting that siRNA precursors are produced by Pol II-mediated bidirectional readthrough transcription of circular viral dsDNA [31,32]. Nonetheless, small amounts of 21 nt begomoviral siRNAs are generated by an RDR6- and DCL4-dependent pathway and those so-called secondary siRNAs are involved in cell-to-cell spread of RNAi [30,31]. In *A*. *thaliana*, the RDRγ genes RDR3, RDR4 and RDR5 are located adjacent to each other and their functionality (if any) in siRNA biogenesis, gene silencing or antiviral defense remains unknown [33]. In rice (*Oryza sativa*), the γ-clade RDR3 is involved in the regulation of transposons and other repeat-rich genomic regions generating 21 and 24 nt siRNAs and biochemical evidence shows its polymerase activities on both ssRNA and ssDNA templates [34] similar to the activities reported for the α-clade RDR1 from tomato [35].

*Solanum lycopersicum* plants infected with TYLCV accumulate 21, 22 and 24 nt siRNAs derived from both strands of the entire virus genome [36–38], indicating that antiviral RNAi is mediated by at least three tomato DCLs. The tomato *Ty-1* gene-encoded RDRγ mediates resistance against TYLCV by enhancing production of virus-derived 22 and 24 nt siRNAs on expense of 21 nt siRNAs [38] and increasing cytosine methylation of viral DNA [39], suggesting its involvement in 24 nt siRNA-directed transcriptional silencing of viral genes and possibly posttranscriptional silencing of viral mRNAs directed by 22 nt siRNAs [38]. This hypothesis is consistent with the findings that the resistance against TYLCV is compromised in *Ty-1* plants co-infected with cucumber mosaic virus or a begomoviral betasatellite, which are known to encode suppressors of posttranscriptional and transcriptional gene silencing [40].

In Morocco, an invasive recombinant between the IL strain of TYLCV (TYLCV-IL) and TYLCSV was detected in 2010 in the *Ty-1* resistant plants exhibiting typical symptoms of TYLCD [41]. In this unusual recombinant, called TYLCV-IS76, a short sequence of the intergenic region of TYLCV-IL between position 1 (the origin of replication and recombination break-point) and position 84 was replaced with the homologous although slightly shorter sequence of TYLCSV (1–76). Extended surveys conducted from 2012 revealed that TYLCV-IS76 had almost totally replaced its parental viruses in the Souss region of Morocco from where it probably originated. Interestingly, the invasion of TYLCV-IS76 coincided with the deployment of *Ty-1* resistant tomato cultivars in this country [41]. Under laboratory conditions, TYLCV-IS76 is positively selected in the *Ty-1* plants where it accumulates at higher levels than its parental viruses and, more intriguing, has a strong deleterious effect on TYLCV-IL, leading to disappearance of this parental virus at late stages of coinfection [42,43]. The molecular mechanisms underlying partial evasion of RDRγ-mediated resistance by TYLCV-IS76 and its strong deleterious impact on TYLCV-IL in mixed infection of *Ty-1* plants are unknown. In this study, we began to uncover these mechanisms by comparative transcriptome and sRNAome profiling of susceptible vs *Ty-1* resistant tomato plants infected with TYLCV-IL, TYLCV-IS76 or combination thereof at early and late stages of infection.

## Results and discussion

We inoculated 14-days old tomato seedlings of a *Ty-1* resistant (R) cultivar (Pristyla) and a nearly isogenic susceptible (S) one with the infectious clones of TYLCV-IL isolate RE4 (AM409201; hereafter IL), TYLCV-IS76 isolate G8 (LN812978; hereafter IS76) or their combination (IL+IS76). Viral DNA loads were measured with quantitative (q)PCR, while loads, production rates and profiles of viral mRNAs and virus-derived siRNAs were analysed by Illumina sequencing of total RNA from systemically infected leaf tissues collected at 10 and 30 days post-inoculation (dpi). Two biological replicates were analysed for each condition.

**Fig 1:**
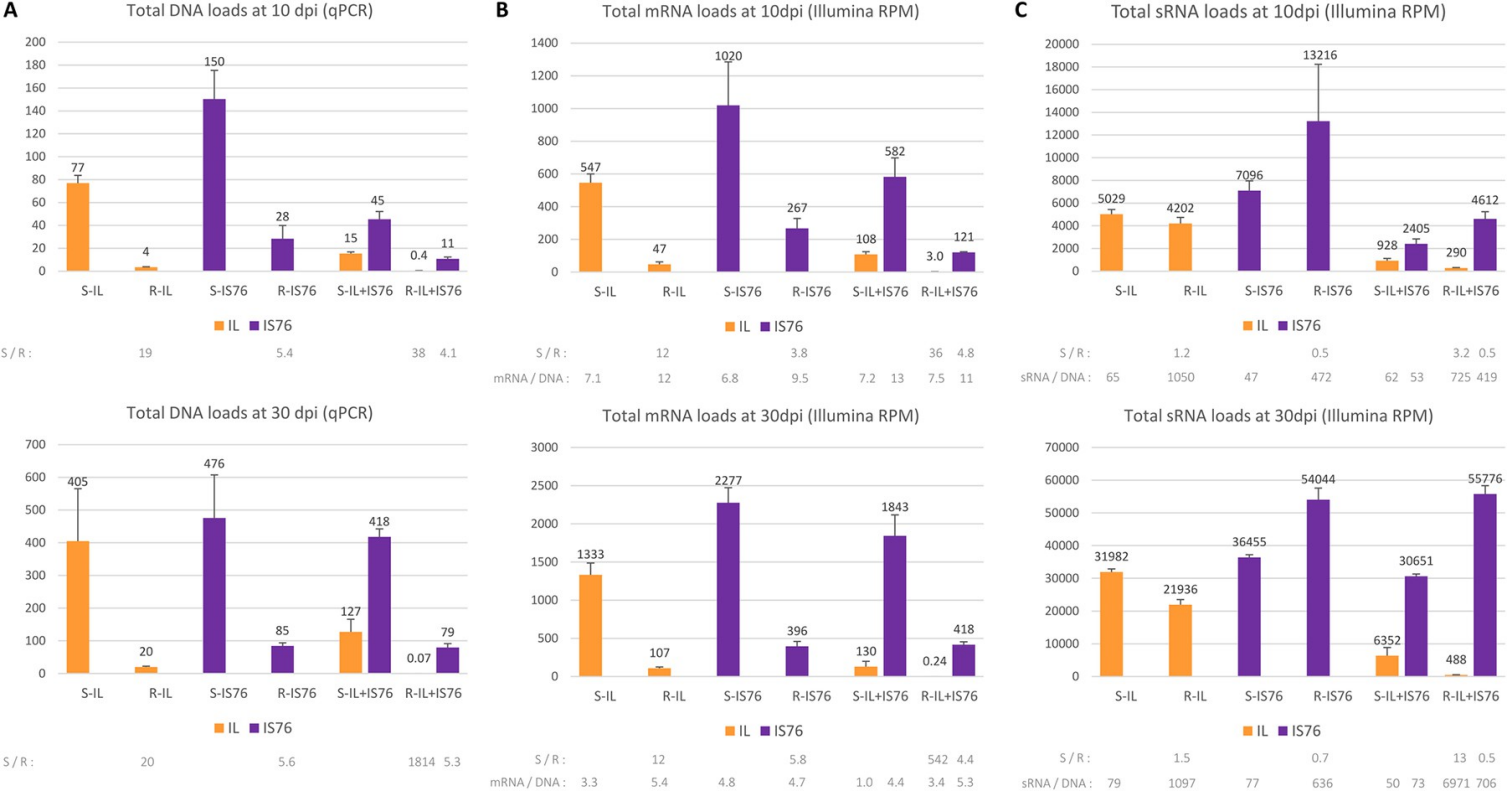
Total viral DNA, mRNA and small (s)RNA accumulation in susceptible (S) and *Ty-1* resistant (R) tomato plants infected with TYLCV-IL, its recombinant derivative TYLCV-IS76, or their combination (IL+IS76) at 10 and 30 days post inoculation (dpi). (**A**) Viral DNA loads measured by quantitative PCR (qPCR). The qPCR data were normalized using the tomato 25S rRNA gene. (**B**) Loads of total viral mRNAs measured by Illumina RNA-seq in reads per million (RPM) of total (plant + viral) mRNA reads. (**C**) Loads of total viral small (s)RNAs measured by Illumina sRNA-seq in reads per million (RPM) of total (plant + viral) sRNA reads in the size range from 15 to 34 nts. In all panels, bar graphs plot the loads for two biological replicates per each condition, with the standard error shown with a capped vertical line and the mean value indicated above. Bars for IS76 and IL are colour-coded in purple and yellow, respectively. Ratios of the mean values for each virus (IL, IS76) and their combination (IL+IS76) in S vs R plants (S/R) are given below each graph. In the case of viral mRNAs and sRNAs, their production rates (the total mRNA or total sRNA load in RPM divided by the respective virus DNA load)—“mRNA/DNA” and “sRNA/DNA”—are also indicated below the graphs.

Consistent with the previous studies [39,41,42], the *Ty-1* resistance gene encoding RDRγ had a negative impact on viral DNA accumulation. Indeed, following single virus infection at both 10 and 30 dpi, the loads of viral DNA in R plants carrying the functional RDRγ were much lower than those in nearly isogenic S plants lacking the functional RDRγ (Fig 1A). Notably, whereas the ratio of viral loads between S and R plants was ~20 for IL, it was only ~5.5 for IS76 at both time points, indicating that the recombinant IS76 was able to evade the defence mediated by RDRγ better than its parent IL. At 10 dpi, the DNA loads were higher for IS76 than IL ~2 times in S plants and ~8 times in R plants. By 30 dpi, IS76 and IL accumulated their DNA at similar levels in S plants, whereas in R plants the DNA loads were ~4 times higher for IS76 (Fig 1A), owing to evasion of RDRγ-mediated resistance.

In mixed infection (IL+IS76) of S and R plants at 10 dpi, the DNA loads of IL were respectively 5.1 and 10 times lower than in singly infected S and R plants, whereas the DNA loads of IS76 were respectively 3.3 and 2.5 times lower than in singly infected S and R plants (Fig 1A). By 30 dpi, the DNA loads of IL were respectively 3.2 and 286 times lower than in singly infected S and R plants. In sharp contrast, the DNA loads of IS76 by 30 dpi were similar between single and mixed infection of both S and R plants (Fig 1A). Thus, IL had only a transient negative impact on IS76 in both S and R plants, whereas IS76 had a strong negative impact on IL at both early and late stages of co-infection in S plants and nearly eliminated IL from co-infected R plants, confirming its remarkable competitiveness in mixed infections [42,43].

### RDRγ modulates production rates of viral mRNAs and strongly enhances production rates of viral sRNAs from both viruses

Using the Illumina sequencing data (S1 and S2 Datasets), we first measured the collective loads of viral mRNAs and viral sRNAs in reads per million (RPM) of total (plant+viral) mRNA and total (plant+viral) sRNA reads, respectively. Whereas viral mRNA loads overall correlated relatively well with viral DNA loads (Fig 1B vs 1A), viral sRNA loads did not correlate with viral DNA loads (Fig 1C vs 1A).

Despite the apparent correlation between viral DNA and mRNA loads, calculation of the production rates of viral mRNAs (i.e., viral mRNA loads divided by viral DNA loads) did reveal differences between S and R plants and between the time-points (Fig 1B, below the graphs).

In single infections at 10 dpi, the viral mRNA production rates (mPR) for IL and IS76 in S plants (mPR = 7.1 and 6.8, respectively) were lower than those in R plants (mPR = 12 and 9.5, respectively). Thus, both viruses appeared to compensate in part the strong negative impact of RDRγ on viral DNA replication by increased production of viral mRNAs. By 30 dpi, the mRNA production rates dropped down for both viruses in both S and R plants and the compensatory effect was observed only for IL (mPR = 3.3 and 5.4, respectively) but not for IS76 (mPR = 4.8 and 4.7, respectively) (Fig 1B).

In mixed infections at 10 dpi, the rates of mRNA production from IL were similar between S and R plants (mPR = 7.2 and 7.5, respectively). Hence, in the presence of IS76, IL was not able to cope with the negative impact of RDRγ by increasing its mRNA production rate. In contrast, the presence of IL did not affect the rates of mRNA production from IS76 which became even higher in both S and R plants (mPR = 13 and 11, respectively), compared to the respective plants singly infected with IS76 (mPR = 6.8 and 9.5, respectively). By 30 dpi, the rates of mRNA production from both viruses dropped down in both S and R plants. Compared to single infections at this time-point, IL only slightly affected IS76 in S plants (mPR = 4.4 vs 4.8) and R plants (mPR = 5.3 vs 4.7). In contrast, IS76 had a substantial negative impact on IL in both S (mPR = 1.0 vs 3.3) and R (mPR = 3.4 vs 5.4) plants (Fig 1B).

The overall production rates of viral sRNAs (i.e., viral sRNA loads in RPM divided by viral DNA loads) in R plants were drastically higher than in S plants, irrespective of the conditions (Fig 1C, below the graphs). Notably, the positive effect of RDRγ on the sRNA production rate (sPR) was more pronounced for IL. Indeed, in single infections at both 10 and 30 dpi the ratio of sPRs in R vs S plants was much higher for IL (1050/65 and 1097/79, respectively), compared to IS76 (472/47 and 636/77). In mixed infections, IL did not have any substantial effect on the rates of sRNA production from IS76. In contrast, IS76 modulated those from IL, with the most pronounced effect observed in R plants at 30 dpi (sPR = 6971 vs 1097 in single infection).

Taken together, RDRγ modulates the overall production rates of viral mRNAs and strongly enhances the overall production rates of viral sRNAs from both viruses in both single and mixed infections at both time-points.

### IS76 undergoes faster transition to overexpression of the rightward genes

**Fig 2:**
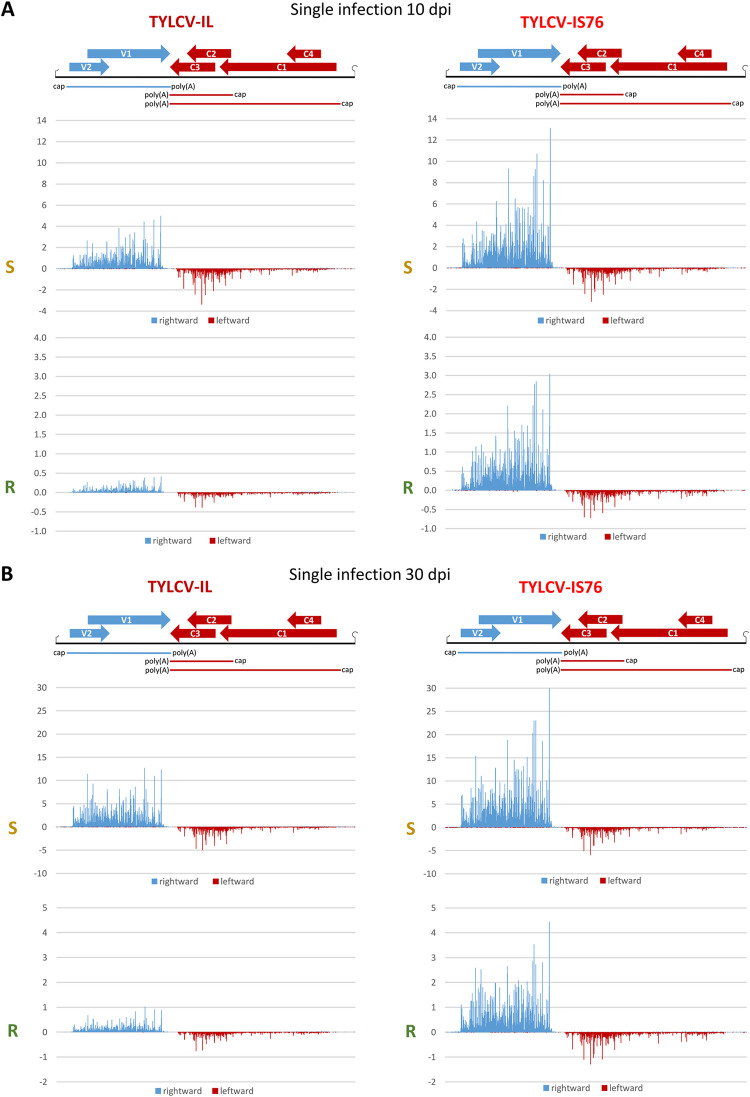
Single-nucleotide resolution maps of viral mRNA reads in susceptible (S) and *Ty-1* resistant (R) tomato plants infected with TYLCV-IL or its recombinant derivative TYLCV-IS76 at 10 (**A**) and 30 (**B**) days post inoculation (dpi). For each condition, Illumina mRNA-seq 100 nt paired-end reads were mapped onto the reference sequences of IL and IS76 genomes with zero mismatches (see S3 Dataset for more details of mapping). Histograms plot the numbers of viral reads at each nucleotide position of the IL and IS76 genomes (2781 and 2773 bp in length, respectively): blue bars above the axis represent virion strand (rightward) reads starting at each respective position, while red bars below the axis represent complementary strand (leftward) reads ending at each respective position. The viral genome organization is shown schematically above the histograms, with ORFs of the viral rightward (V1, V2) and leftward (C1-to-C4) genes shown with blue and red arrows, respectively, and capped and polyadenylated viral mRNAs (V2-V1, C1-C4 and C2-C3) shown as solid blue and red lines.

**Fig 3:**
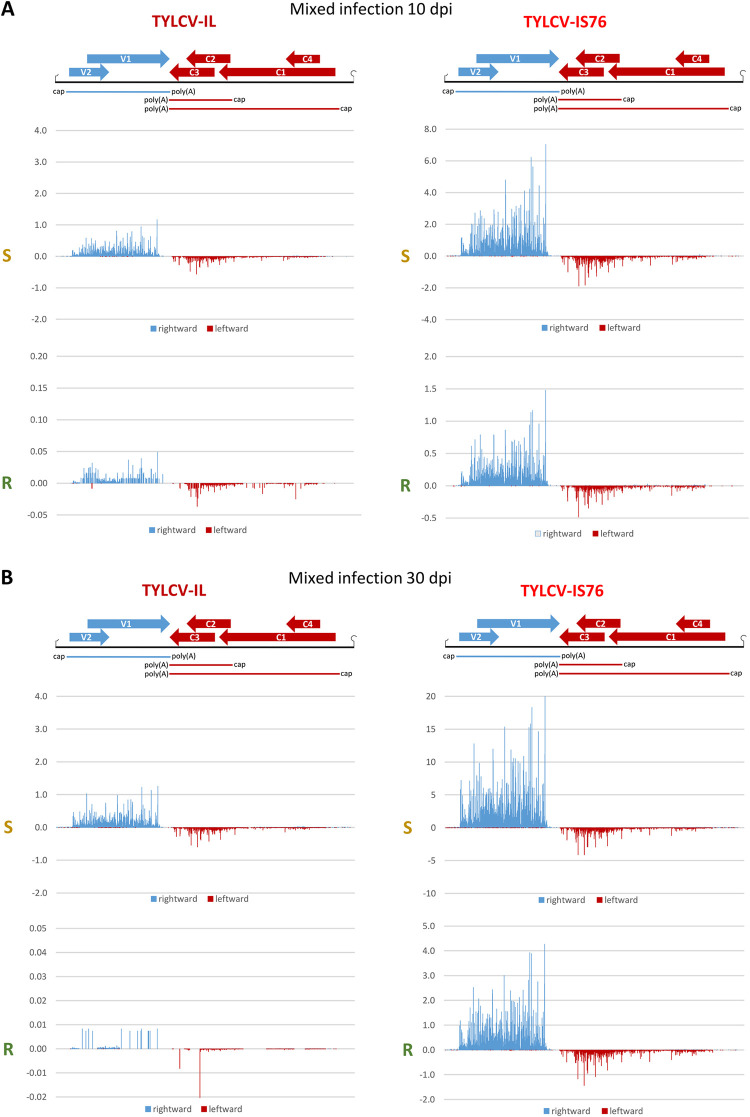
Single-nucleotide resolution maps of viral mRNA reads in susceptible (S) and *Ty-1* resistant (R) tomato plants co-infected with TYLCV-IL and its recombinant derivative TYLCV-IS76 at 10 (**A**) and 30 (**B**) days post inoculation (dpi). For each condition, Illumina 100 nt paired-end reads were mapped onto the reference sequences of IL and IS76 genomes with zero mismatches (see S3 Dataset for more details of mapping). Histograms plot the numbers of viral reads at each nucleotide position of the IL and IS76 genomes (2781 and 2773 bp in length, respectively): blue bars above the axis represent virion strand (rightward) reads starting at each respective position, while red bars below the axis represent complementary strand (leftward) reads ending at each respective position. The viral genome organization is shown schematically above the histograms, with ORFs of the viral rightward (V1, V2) and leftward (C1-to-C4) genes shown with blue and red arrows, respectively, and capped and polyadenylated viral mRNAs (V2-V1, C1-C4 and C2-C3) shown as solid blue and red lines.

Mapping of Illumina mRNA-seq 100 nt paired-end reads on the reference genomes of IL and IS76 revealed the three Pol II transcription units previously reported for begomoviruses, one rightward (virion strand) unit for the V2-V1 mRNA and two leftward (complementary strand) units for 3’-coterminal C1-C4 and C2-C3 mRNAs (Figs 2 and 3 and S3 Dataset). Consistent with a previous mRNA-seq study of TYLCV [37], reads were not homogeneously distributed along the length of each mRNA, due to sequence-specific biases in Illumina library preparation and sequencing protocols leading to either underrepresentation or overrepresentation of certain sequences. Indeed, the map patterns are similar for each viral mRNA between S and R plants and between 10 and 30 dpi. The relative abundance of all reads (in RPM) representing each viral mRNA differed substantially, showing that V2-V1 mRNA is the most abundant for both viruses in all conditions, followed by the second most abundant C2-C3 mRNA and the least abundant C1-C4 mRNA (Figs 2, 3 and S1). Similar mRNA profiles were observed for TYLCV-IL (isolate Almeria) in susceptible tomato (cv. Moneymaker) at 7, 14 and 21 dpi [37].

**Fig 4:**
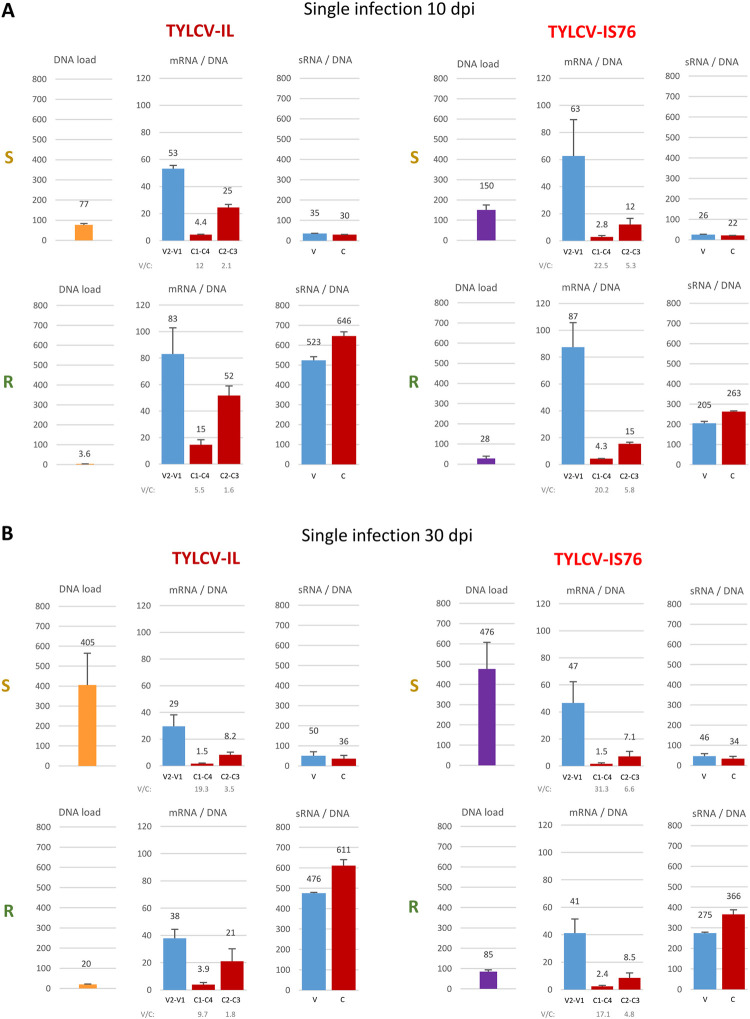
Production rates of viral mRNAs and sRNAs derived from virion (V) and complementary (C) stands of the viral genome in susceptible (S) and *Ty-1* resistant (R) tomato plants singly infected with TYLCV-IL and its recombinant derivative TYLCV-IS76 at 10 (**A**) and 30 (**B**) days post inoculation (dpi). Illumina mRNA-seq reads representing each mRNA (V2-V1, C1-C4, C2-C3) of IL and IS76 and Illumina sRNA-seq reads (in size range from 20 to 25 nts) representing virion and complementary strands of each virus genome were counted in reads per million (RPM) of total (plant + viral) reads. The resulting counts were divided by the load of respective viral DNA measured by qPCR and, in the case of viral mRNA, by the length of each transcription unit in nucleotides. In each panel, bar graphs plot loads of the viral DNA (yellow and purple bars for IL and IS76, respectively) and the production rates of the rightward (V2-V1) and leftward (C1-C4, C2-C3) mRNAs (blue and red bars, respectively) and the sRNAs derived from the virion and complementary stands (blue and red bars, respectively). In all cases, the loads are for two biological replicates per each condition, with the standard error shown with a capped vertical line and the mean value indicated above.

**Fig 5:**
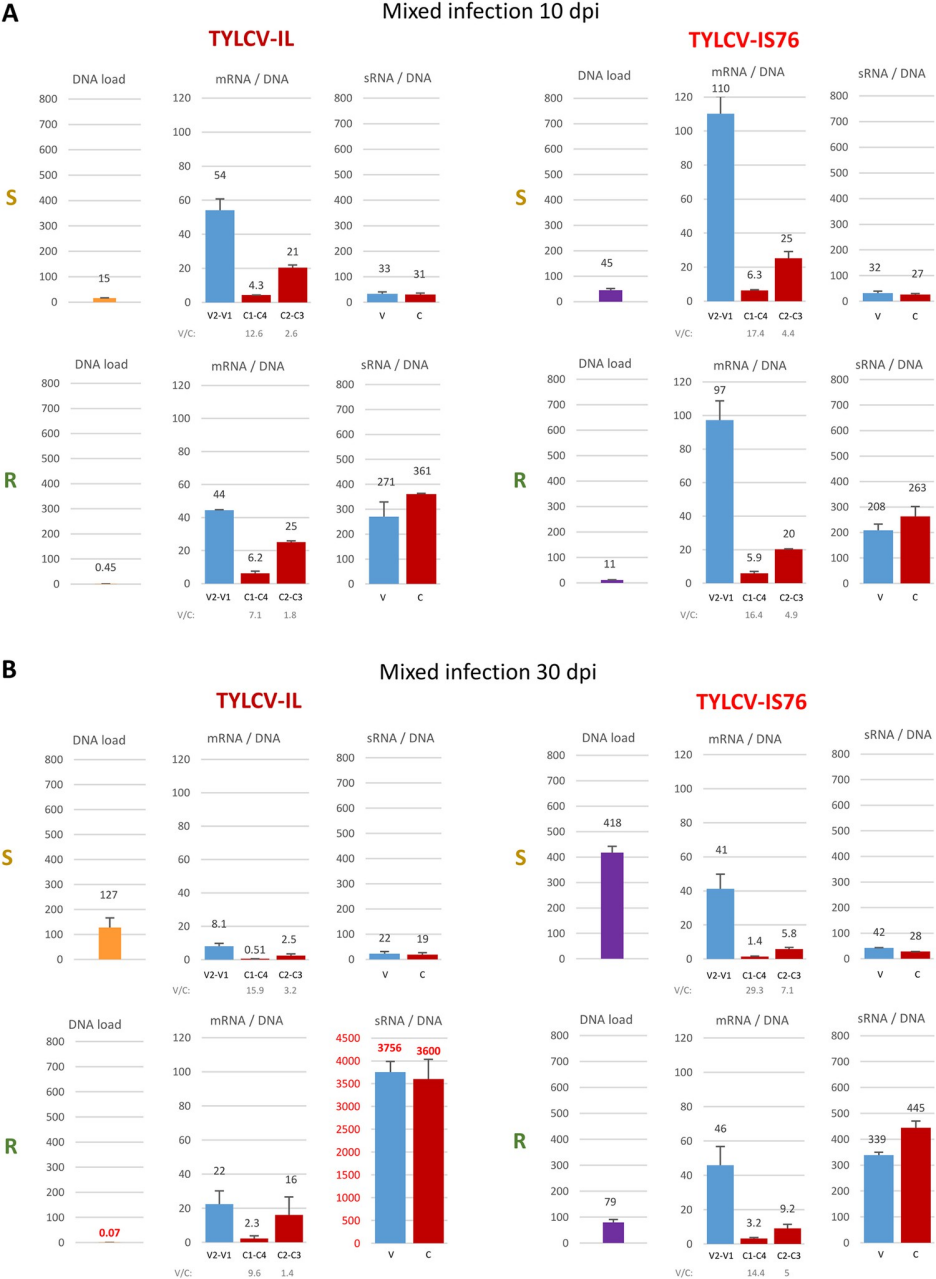
Production rates of viral mRNAs and sRNAs derived from virion (V) and complementary (C) stands of the viral genome in susceptible (S) and *Ty-1* resistant (R) tomato plants co-infected with TYLCV-IL and its recombinant derivative TYLCV-IS76 at 10 (**A**) and 30 (**B**) days post inoculation (dpi). Illumina mRNA-seq reads representing each mRNA (V2-V1, C1-C4, C2-C3) of IL and IS76 and Illumina sRNA-seq reads (in size range from 20 to 25 nts) representing virion and complementary strands of each virus genome were counted in reads per million (RPM) of total (plant + viral) reads. The resulting counts were divided by the load of respective viral DNA measured by qPCR and, in the case of viral mRNA, by the length of each transcription unit in nucleotides. In each panel, bar graphs plot loads of the viral DNA (yellow and purple bars for IL and IS76, respectively) and the production rates of the rightward (V2-V1) and leftward (C1-C4, C2-C3) mRNAs (blue and red bars, respectively) and the sRNAs derived from the virion and complementary stands (blue and red bars, respectively). In all cases, the loads are for two biological replicates per each condition, with the standard error shown with a capped vertical line and the mean value indicated above.

The most striking difference between IS76 and IL is that in all conditions IS76 accumulates its V2-V1 mRNA at relatively higher levels, compared to IL (Figs 2, 3 and S1). This suggests that the recombination region of IS76 modulates the bidirectional promoter in favour of rightward transcription. To estimate the activities of the bidirectional promoter driving leftward transcription of C1-C4 mRNA and rightward transcription of V2-V1 mRNA as well as the monodirectional promoter driving transcription of C2-C3 mRNA, we calculated the production rate of each viral mRNA (i.e., the mRNA load in RPM divided by the mRNA transcription unit length in nucleotides and by the viral DNA load). The results revealed that the ratio of production rates of V2-V1 mRNA vs C1-C4 mRNA was higher for IS76 than IL in all conditions (Figs 4 and 5). The higher ratio of rightward-to-leftward transcription rates may reflect a faster replication cycle of IS76 in which encapsidation of viral DNA by the viral CP (translated from V2-V1 mRNA) begins earlier than for IL. This hypothesis is consistent with the accumulation dynamics of viral DNA: IS76 accumulates (and hence replicates) its DNA faster than IL in both S and R plants (Fig 1A).

Interestingly, in singly infected plants at 10 dpi the ratio of production rates of V2-V1 mRNA vs C1-C4 mRNA for IL is 2.2 times lower in R plants than in S plants (mPR ratio = 5.5 vs 12), whereas this ratio is almost unaffected for IS76 (mPR ratio = 20.2 vs 22.5). Thus, RDRγ has a negative impact on the replication cycle of IL by hampering the transition from leftward to rightward transcription.

The transition from leftward to rightward transcription is controlled by the intergenic region cis-elements, most notably those in the rightward promoter transactivated by the viral TrAP [44,45]. Additionally the short iterated sequence repeats (iterons) in the leftward promoter regulate this transition as the viral Rep binds these iterons and thereby inhibits leftward transcription of its own mRNA as demonstrated for a bipartite begomovirus [22]. Finally, as shown for another bipartite begomovirus [21], Rep-mediated inhibition of the leftward transcription further enhances the rightward transcription of V2-V1 mRNA transactivated by TrAP/C2 and thereby promotes overexpression of the viral CP required for encapsidation.

Notably, the production rates of C1-C4 mRNA from both viruses in all conditions correlated with those of C2-C3 mRNA (Fig 4). Indeed, the C2-C3/C1-C4 ratios are all within a relatively narrow range (3.5 to 5.7), suggesting that C1-C4 mRNA transcription driven by the bidirectional promoter and C2-C3 mRNA transcription driven by the downstream monodirectional promoter are co-regulated. The mechanism of this co-regulation remains to be investigated.

Collectively, the results reveal a mechanism explaining how IS76 copes better than IL with the negative impact of RDRγ on virus replication. While RDRγ slows down the transition of IL to the cell infection stage favouring the rightward transcription over the leftward transcription, IS76 undergoes this transition almost as fast as in the absence of functional RDRγ.

It is worth noting that the V2 ORF-encoded protein is a strong suppressor of antiviral RNAi and gene silencing [5–11]. Favouring expression of this protein via enhanced transcription of V2-V1 mRNA at earlier stages of cell infection would allow the recombinant IS76 to better suppress antiviral RNAi and, in particular, to counteract the repressive viral sRNAs whose production rates are strongly enhanced by RDRγ for both viruses at both 10 and 30 dpi (Fig 1C).

Our assertions that IS76 replicates faster than IL and that RDRγ slows down viral replication, which are based on the differences in viral DNA accumulation dynamics and leftward-to-rightward transcription rate ratios, were also supported by our Southern blot hybridization analysis of viral DNA forms with strand-specific probes. Indeed, in S plants at 30 dpi, when the dsDNA intermediates of replication were above the detection threshold for both viruses and accumulated at comparable levels, circular ssDNA products of rolling-circle replication accumulated at higher levels for IS76 than IL (S2 Fig). Furthermore, the ssDNA-to-dsDNA ratio of IS76 was higher in S plants, compared to R plants at this time point (S2D Fig).

### IS76 is more transcriptionally active and reduces the transcription rates of all IL mRNAs in the course of coinfection

In coinfected S plants at 10 dpi, the production rates of all mRNAs from IL were similar to those observed in S plants singly infected with IL, while the presence of IL resulted in a ~2 times increase in the production rate of each mRNA of IS76 (Figs 5 vs 4). On the other hand, in coinfected R plants at 10 dpi the individual production rates of IS76 mRNAs were only slightly higher than those observed in R plants singly infected with IS76, and the presence of IS76 resulted in a ~2 times decrease in the individual production rate of each IL mRNA (Figs 5 vs 4). By 30 dpi the negative effect of IS76 on mRNA production from IL becomes evident in both S and R plants. In coinfected S plants, the production rates of all IL mRNAs were proportionally decreased, each about 3 times, compared to singly infected S plants (Figs 5 vs 4). In contrast, the individual mRNA production rates of IS76 were comparable in singly infected and coinfected S plants at 30 dpi. In coinfected R plants, where only residual amounts of IL DNA were detected by 30 dpi, the individual production rates of IS76 mRNAs were comparable to those observed in singly infected R plants and DNA accumulation of IS76 almost reached the levels observed in singly infected R plants. On the other hand, the presence of IS76 reduced the individual production rates of all IL mRNAs in R plants, although to a lesser extent than in S plants.

Notably, mixed infection did not have any drastic effect on the rightward-to-leftward mRNA production rate ratio of IL or IS76 (Figs 5 vs 4).

### RDRγ boosts the production rates of viral siRNAs from both strands of the entire virus genome, with the most pronounced effects at the promoter and terminator regions of both viruses

**Fig 6:**
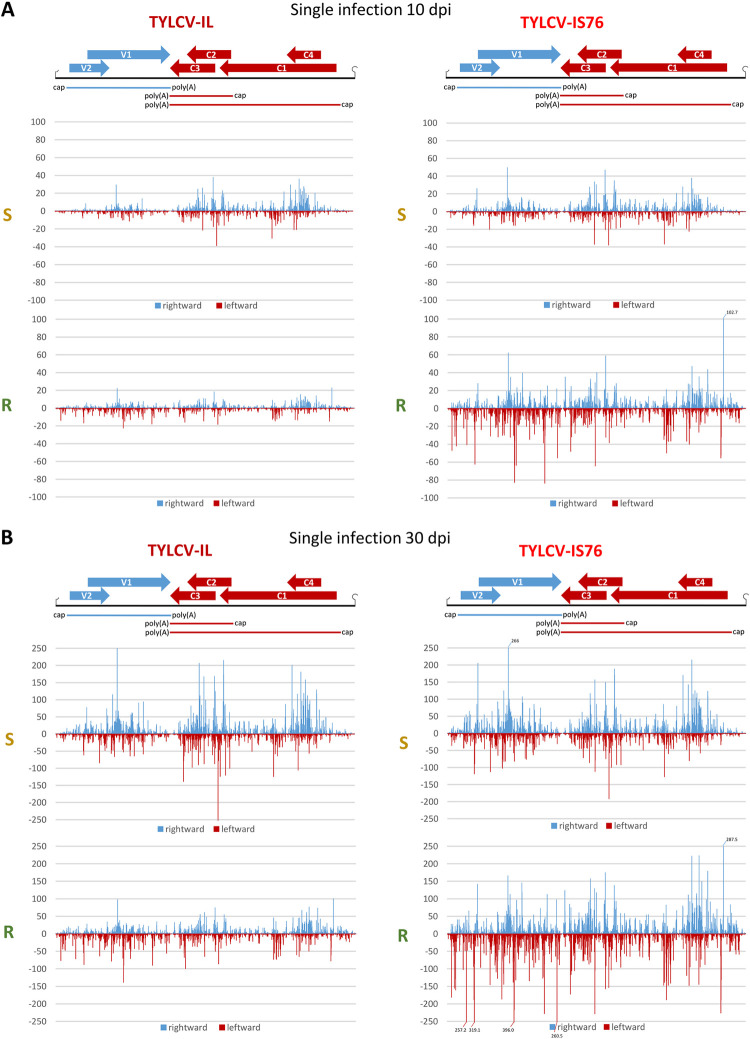
Single-nucleotide resolution maps of 20–25 nt viral small (s)RNAs in susceptible (S) and *Ty-1* resistant (R) tomato plants infected with TYLCV-IL or its recombinant derivative TYLCV-IS76 at 10 (**A**) and 30 (**B**) days post inoculation (dpi). For each condition, Illumina sRNA-seq reads in the size range from 20 to 25 nts were mapped onto the reference sequences of IL and IS76 genomes with zero mismatches (see S4 Dataset for more details of mapping and maps of each size class of viral sRNAs). Histograms plot the numbers of viral reads at each nucleotide position of the IL and IS76 genomes (2781 and 2773 bp in length, respectively): blue bars above the axis represent virion strand (rightward) reads starting at each respective position, while red bars below the axis represent complementary strand (leftward) reads ending at each respective position. The viral genome organization is shown schematically above the histograms, with ORFs of the viral rightward (V1, V2) and leftward (C1-to-C4) genes shown with blue and red arrows, respectively, and capped and polyadenylated viral mRNAs (V2-V1, C1-C4, C2-C3) shown as solid blue and red lines.

**Fig 7:**
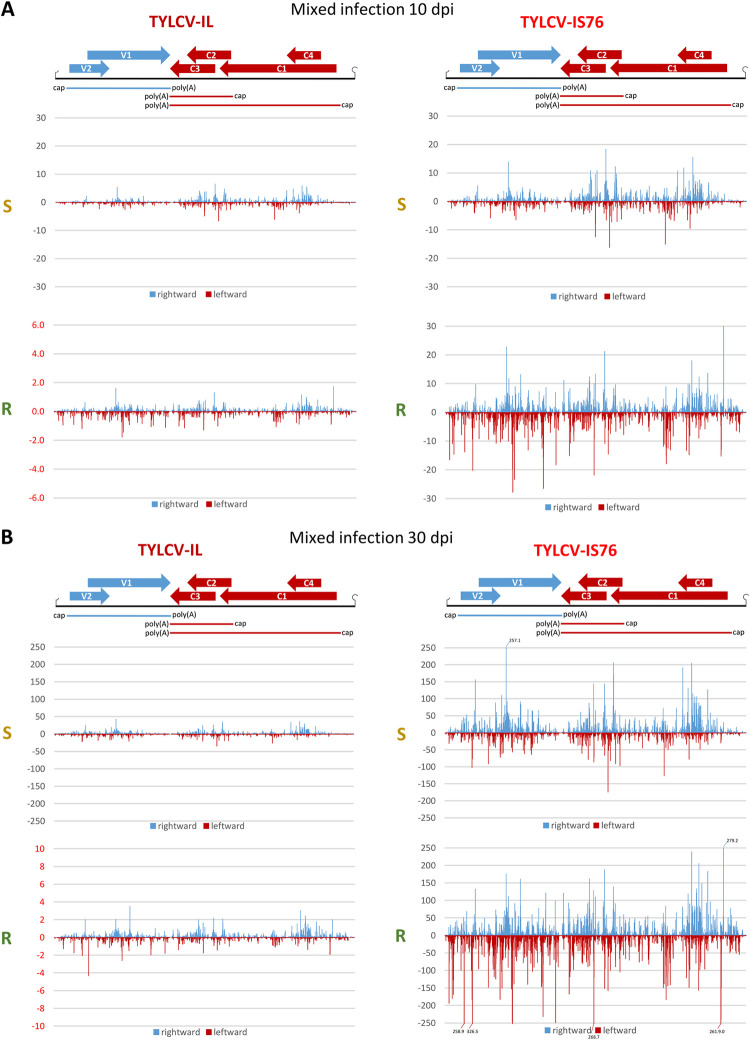
Single-nucleotide resolution maps of 20–25 nt viral small (s)RNAs in susceptible (S) and *Ty-1* resistant (R) tomato plants co-infected with TYLCV-IL and its recombinant derivative TYLCV-IS76 at 10 (**A**) and 30 (**B**) days post inoculation (dpi). For each condition, Illumina sRNA-seq reads in the size range from 20 to 25 nts were mapped onto the reference sequences of IL and IS76 genomes with zero mismatches (see S4 Dataset for details of read mapping and counting in mixed infection and for maps of each size class of viral sRNAs). Histograms plot the numbers of viral reads at each nucleotide position of the IL and IS76 genomes (2781 and 2773 bp in length, respectively): blue bars above the axis represent virion strand (rightward) reads starting at each respective position, while red bars below the axis represent complementary strand (leftward) reads ending at each respective position. The viral genome organization is shown schematically above the histograms, with ORFs of the viral rightward (V1, V2) and leftward (C1-to-C4) genes shown with blue and red arrows, respectively, and capped and polyadenylated viral mRNAs (V2-V1, C1-C4, C2-C3) shown as solid blue and red lines.

**Fig 8:**
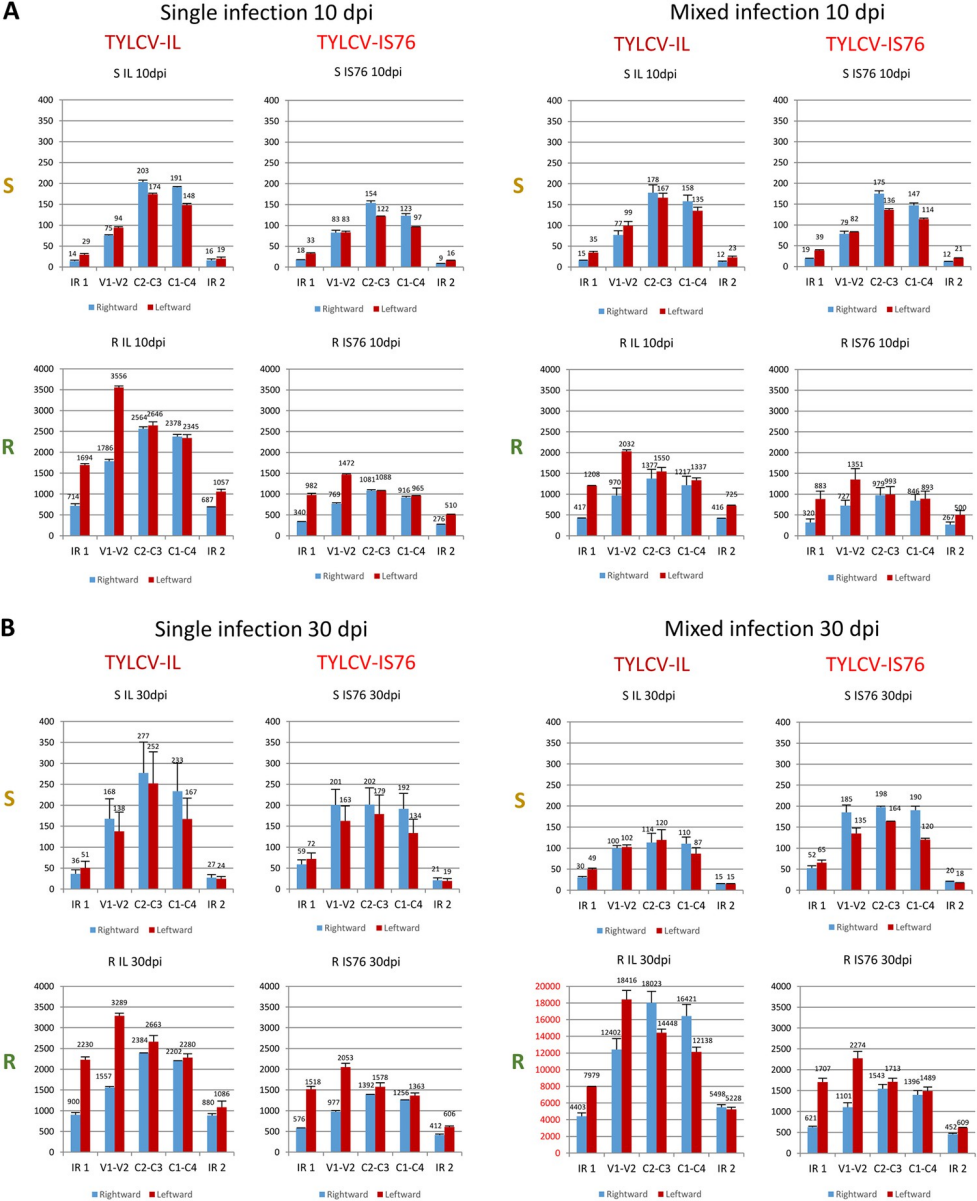
Production rates of sRNAs from different regions of virion (V) and complementary (C) stands of the viral genome in susceptible (S) and *Ty-1* resistant (R) tomato plants infected with TYLCV-IL, its recombinant derivative TYLCV-IS76 or a combination thereof (IL+S76) at 10 (**A**) and 30 (**B**) days post inoculation (dpi). Illumina sRNA-seq reads in the size range from 20 to 25 nts representing virion and complementary strands of each transcription unit (V2-V1, C1-C4, C2-C3) and two parts of the intergenic region with the rightward (IR1) and the rightward (IR2) promoters of IL and IS76 genomes were counted in reads per million (RPM) of total (plant + viral) reads (see Material and Methods for further details of read counting in mixed infection). The resulting counts were divided by the length of each region in nucleotides and the load of respective viral DNA measured by qPCR and then multiplied by 10000. Bar graphs plot sRNA loads for the rightward (V2-V1) and leftward (C1-C4, C2-C3) mRNA transcription units and two parts of the intergenic region (IR1 and IR2). The loads of sRNAs derived from the virion and complementary stands of each region of the viral genome are represented with blue and red bars, respectively. In all cases, the loads are for two biological replicates per each condition, with the standard error shown with a capped vertical line and the mean value indicated above.

**Fig 9:**
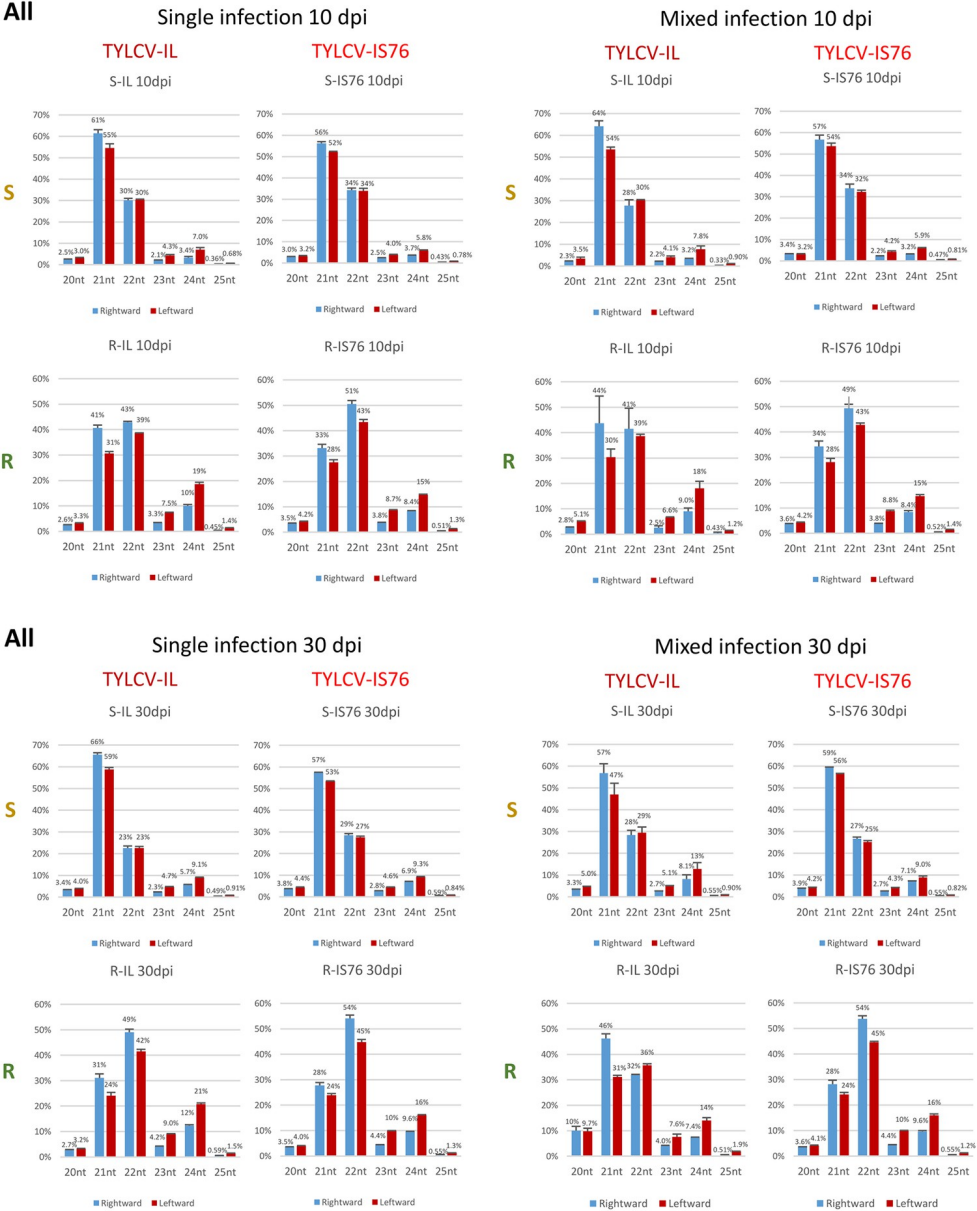
Size profiles of viral sRNAs derived from the complete viral genome in susceptible (S) and *Ty-1* resistant (R) tomato plants infected with TYLCV-IL, its recombinant derivative TYLCV-IS76 or a combination thereof (IL+S76) at 10 and 30 days post inoculation (dpi). Illumina sRNA-seq reads in the size range from 20 to 25 nts mapped to the virion (rightward) and complementary (leftward) strands of the complete viral genome were counted and percentages (%) of 6 individual size-classes in the total 20–25 nt viral reads (set to 100%) were calculated and plotted as bar graphs, with blue and red bars representing rightward and leftward strands, respectively. In all panels, the percentages are for two biological replicates per each condition, with the standard error shown with a capped vertical line and the mean value indicated above.

Mapping Illumina sRNA reads on the reference genomes of IL and IS76 revealed that viral sRNAs are derived from both strands of the entire virus genomes in both S and R plants and at both time-points (Figs 6, 7, 8 and S3 and S4 Dataset) and are dominated by the three major size-classes (Fig 9 and S4 Dataset; see below). In both singly infected and coinfected S plants at both 10 and 30 dpi, the hotspots of viral sRNAs of sense and antisense polarities are concentrated within the Pol II transcription units and are underrepresented within the intergenic region (IR) with bidirectional promoter, the poly(A) sites-containing Pol II terminator region and, to a lesser extent, the C2-C3 promoter region (between C2 and C4 ORFs). By contrast in R plants singly infected or coinfected with IL and IS76 at both 10 and 30 dpi, the viral sRNA hotspots are more evenly distributed along the entire virus genome including the IR and the terminator region (Figs 6 and 7). These results are generally consistent with the previous studies profiling TYLCV-IL sRNAs at different time points in susceptible tomato plants [36,37] and comparing the sRNA profiles of TYLCV-IL (isolate Almeria) in susceptible (cv. Moneymaker) and *Ty-1* resistant (cv. Tygress) plants [38].

Production of viral sRNAs from both strands of the entire virus genome including the “non-transcribed” IR (between the Pol II transcription start sites of the leftward C1-C4 and the rightward V2-V1 units) suggests that dsRNA precursors of viral siRNAs are generated by Pol II-mediated readthrough transcription far beyond the poly(A) signals in both leftward and rightward directions as proposed earlier for a bipartite begomovirus [30,31]. In support of this hypothesis, our Illumina sequencing analysis of both IL- and IS76-infected plants revealed low-abundance long RNA reads covering the antisense strands of the rightward and leftward transcription units and both strands of the IR, which likely represent remnants of the presumptive readthrough transcripts (S1 Fig and S3 Dataset). The leftward and rightward readthrough transcripts that cover the entire circular virus genome might form dsRNA precursors of viral sRNAs by pairing to the mRNAs of opposite polarity and/or to each other. Only the latter events might generate dsRNA precursors of viral sRNAs derived from the IR outside of the Pol II transcription units. The enrichment of viral sRNAs of sense and antisense polarities in the IRs of both viruses in R plants, compared to S plants, suggests that RDRγ might indirectly promote bidirectional readthrough transcription. Additionally or alternatively, RDRγ might convert readthrough transcripts into dsRNA. In S plants, dsRNA precursors of IR-derived sRNAs might be produced by the α-clade RDRs (RDR1, RDR2 and/or RDR6) using ssRNA substrates generated by Pol II readthrough transcription or by Pol V or Pol IV transcription. Nonetheless, potential activities of the α-clade RDRs cannot explain the viral sRNA hotspots concentrating in the mRNA transcription units. Indeed, Illumina sequencing of viral sRNAs from *A*. *thaliana* wild type and *RDR1/RDR2/RDR6* triple mutant plants infected with a bipartite begomovirus revealed no substantial differences in the viral sRNA accumulation, size, polarity or hotspot profiles [31]. Notably, the latter profiles resemble the respective profiles we observed for IL and IS76 in S plants in that the IR and the Pol II terminator region are depleted in sRNA hotspots.

Next, we calculated the production rates of sRNAs derived from each strand of the Pol II transcription units and two parts of the IR containing the rightward (IR1) and the leftward (IR2) promoter elements. To this end, counts (in RPM) of sRNA reads derived from each strand of the respective regions were divided by the length of the respective regions in nucleotides and then by the viral DNA load. Comparison of the resulting sRNA production rates revealed their dramatic increase in R plants, compared to S plants, for both viruses in both single and mixed infections at both time-points (Fig 8), indicating that RDRγ-dependent enhancement of viral sRNA production affects all the regions and strands of the virus genome. Strikingly, this enhancement was much more pronounced in both parts and both strands of the IR than in the Pol II transcription units (Fig 8), consistent with the observed differences in sRNA single-nucleotide resolution maps (Figs 6 and 7).

### Production rates of viral sRNAs from transcription units or IR do not correlate with production rates of viral mRNAs

In singly infected S plants at 10 dpi, the highest sRNA production rates were observed in the C2-C3 and C1-C4 units, followed by the V2-V1 unit, without any substantial forward or reverse strand biases (Fig 8A). However, while the V2-V1 unit produced sRNAs at similar rates in IL- vs IS76-infected S plants, both C1-C4 and C2-C3 units of IL produced sRNAs at higher rates than those of IS76. This coincided with similar production rates of V2-V1 mRNA and higher production rates of both C1-C4 and C2-C3 mRNAs in IL-infected plants (Fig 4). Thus, viral sRNAs of both polarities produced at higher rates from the leftward transcription units do not reduce (but rather increase) the production rates of C1-C4 or C2-C3 mRNAs which can potentially be targeted for cleavage and degradation by the sRNAs of opposite polarity. Moreover, for both IL and IS76 the viral sRNAs of both polarities were produced at similar rates from the C1-C4 and the C2-C3 units that produced their respective mRNAs at drastically different rates. Furthermore, the production rates of sRNAs from both parts of the IR were comparable in S plants singly infected with IL or IS76 (Fig 8A). Since the rate of the rightward V2-V1 mRNA transcription is much higher than that of the leftward C1-C4 mRNA transcription (Fig 4), viral sRNAs derived from the IR-based bidirectional promoter do not appear to regulate Pol II-mediated bidirectional transcription.

By 30 dpi in singly infected S plants, the rates of viral sRNA production were increased for both viruses. This increase was almost proportional (~1.5 times) for each region of the IL genome in each polarity, whereas it was disproportional for IS76 in which both the IR and the V2-V1 unit produced sRNAs at ~2–3 times higher rates, while its leftward units at ~1.2–1.5 times higher rates (Fig 8B vs 8A). Similar to 10 dpi, at 30 dpi the relative production rates of viral sRNAs from different regions of IL and IS76 (Fig 8B) do not correlate with the relative production rates of respective viral mRNAs (Fig 4B). Nonetheless, the overall increase in viral sRNA production rates between 10 and 30 dpi does coincide with the overall decrease in viral mRNA production rates. Following our hypothesis, increased production of viral sRNAs at later stages of cell infection in S plants might be due to increased readthrough transcription generating sRNA precursors.

In singly-infected R plants at 10 dpi, the production rates of viral sRNAs derived from all regions of the IS76 genome were almost proportionally (~2-to-2.5 times) lower than those of the IL genome, indicating that IS76 can better evade the RDRγ activity boosting sRNA production. In contrast to S plants, both viruses produced relatively more abundant sRNAs from the complementary (reverse) strand of the viral genome, especially within the V2-V1 unit and both parts of the IR (Fig 8A). Similar alterations in sRNA hotspot profiles and strand bias were observed previously by Voorburg et al. [38] in TYCLV-infected *Ty-1* resistant vs susceptible plants. Notably, the production rates of V2-V1 mRNAs were comparable between IL and IS76 (Fig 4A), despite 2–2.5-times difference in the production rates of viral sRNAs targeting the V2-V1 promoter and the V2-V1 mRNA. Furthermore, much higher production rates of viral sRNAs from the leftward promoter and leftward transcription units of IL coincided with much higher production rates of the leftward mRNAs, and vice versa for IS76.

By 30 dpi in R plants singly infected with IL, the rates of sRNA production slightly increased in both strands of the IR, while they were slightly decreased in both strands of all the three transcription units (Fig 8). At the same time, production rates of all IL mRNAs were strongly and almost proportionally (2–3 times) decreased (Fig 4B). In the case of IS76, the sRNA production rates were increased almost proportionally in both strands of each region of the virus genome but still remained lower than those in the respective regions and strands of the IL genome. This increase did coincide with decreased production rates of all viral mRNAs (Fig 4B). The sRNA strand biases observed at 10 dpi for both viruses in R plants (see above) were also observed at 30 dpi.

### Negative impact of IS76 on IL in mixed infection can potentially be reinforced by IS76-derived siRNAs

Compared to single infection, the production rates of sRNAs from any region or strand of the IS76 genome did not differ substantially in the presence of IL at any time point of coinfection of S or R plants (Fig 8). At the same time, the production rates of all IS76 mRNAs were higher in the presence of IL in both S and R plants at the early time-point when IL did interfere with IS76 replication (Fig 5). Conversely, IS76 had strong and contrasting effects on sRNA production rates from the IL genome in S vs R plants and at 10 vs 30 dpi. Indeed, in coinfected S plants by 30 dpi the rates of sRNA production from all regions and strands of the IL genome were substantially lower, except for the part of IR with the rightward promoter where the rates were very similar in single and mixed infection. These alterations coincided with a strong decrease in production rates of all IL mRNAs. In sharp contrast, by 30 dpi in coinfected R plants, the rates of sRNA production from all the regions and strands of the IL genome were much higher than in singly infected R plants (Fig 8), which also coincided with a decrease in production rates of all IL mRNAs. The concomitant dramatic decrease of IL DNA accumulation in the presence of IS76 indicates that IL could not cope with the RDRγ-mediated boost in sRNA production from all regions and strands of the viral genome. In addition to IL genome-derived sRNAs, the highly abundant sRNAs derived from all regions of the IS76 genome (Figs 7 and 8) could further repress IL gene expression, because most of them share 100% identity with the IL genome within the transcription units and the IR outside of the recombination region (see S5 Dataset). This cross-target repression is consistent with lower production rates of all IL mRNAs in mixed infection, compared to single infection of both S and R plants. Reciprocal targeting of IS76 gene expression by IL-derived sRNAs is also possible, especially at the early stages of infection of S plants when those sRNAs accumulate at relatively high levels. However, IS76 appears to evade the repressive sRNAs much better than IL, keeping production rates of all mRNAs even higher than in single infection.

### RDRγ boosts the production rates of all three functional size-classes of viral siRNAs and modulates Dicer activities in favour of 22 and 24 nt siRNAs

Consistent with previous studies of TYLCV-IL [36–38] and other begomoviruses (e.g., [31]), the three major (and functional) size-classes of viral siRNAs (21, 22 and 24 nt) derived from both strands of the IL and IS76 genomes were observed in all conditions (Fig 9 and S4 Dataset). Their relative abundance differed substantially between S and R plants. In S plants, the 21 nt class was dominant for both viruses at both 10 and 30 dpi, followed by the second most abundant 22 nt class and much less abundant 24 nt class. In R plants in all conditions, the relative proportions of 22 and 24 nt siRNAs of both polarities were substantially higher than in S plants (Fig 9). A similar difference in viral sRNA size profiles between susceptible and *Ty-1* resistant plants infected with TYLCV-IL has been reported by Voorburg et al. [38].

**Fig 10:**
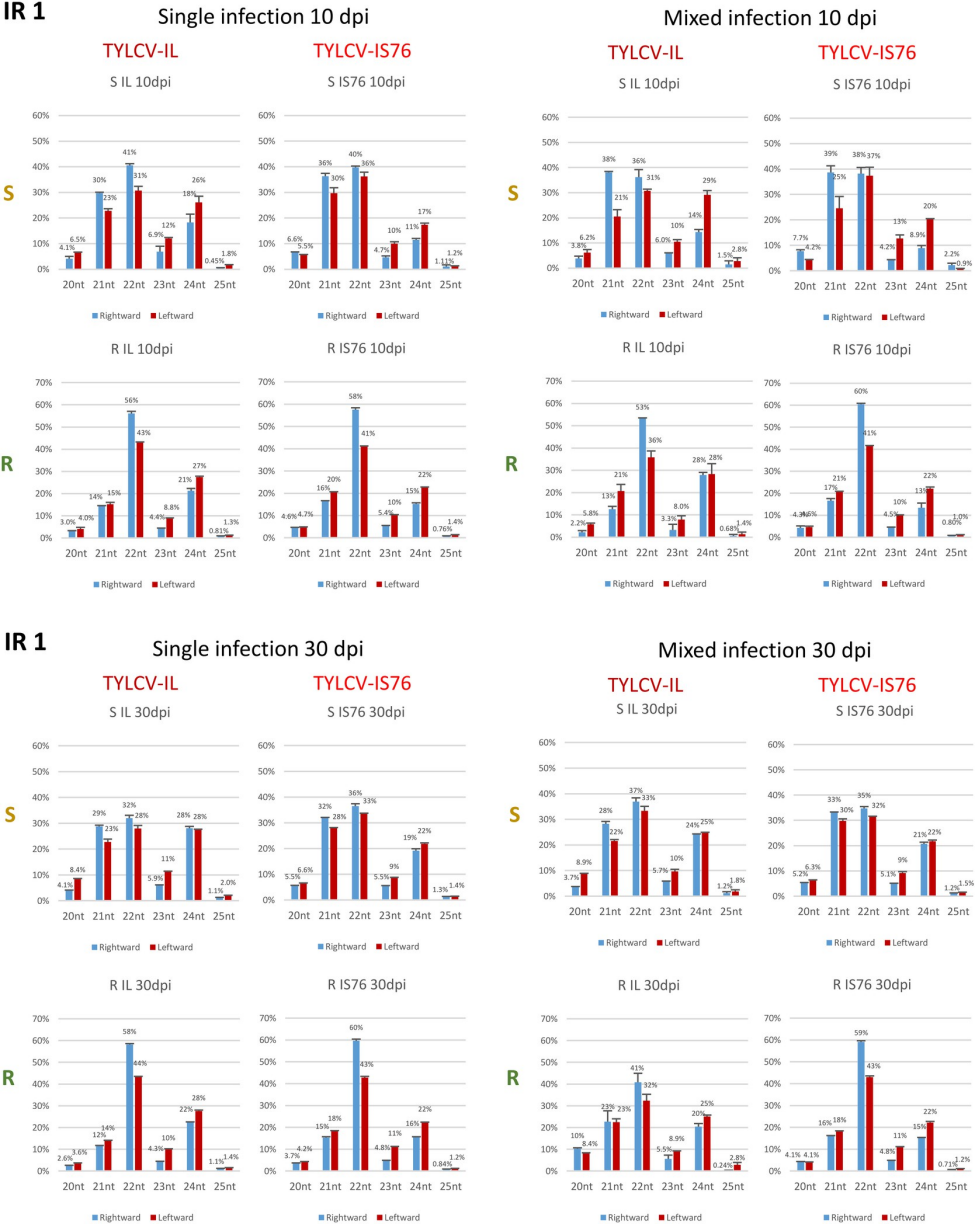
Size profiles of viral sRNAs derived from the rightward promoter-containing intergenic region 1 (IR1) in susceptible (S) and *Ty-1* resistant (R) tomato plants infected with TYLCV-IL, its recombinant derivative TYLCV-IS76 or a combination thereof (IL+S76) at 10 and 30 days post inoculation (dpi). Illumina sRNA-seq reads in the size range from 20 to 25 nts mapped to the viral genome the virion (rightward) and complementary (leftward) strands of the IR1 were counted and percentages (%) of 6 individual size-classes in the total 20–25 nt viral reads (set to 100%) were calculated and plotted as bar graphs, with blue and red bars representing rightward and leftward strands, respectively. In all panels, the percentages are for two biological replicates per each condition with the standard error shown with a capped vertical line and the mean value indicated above.

**Fig 11:**
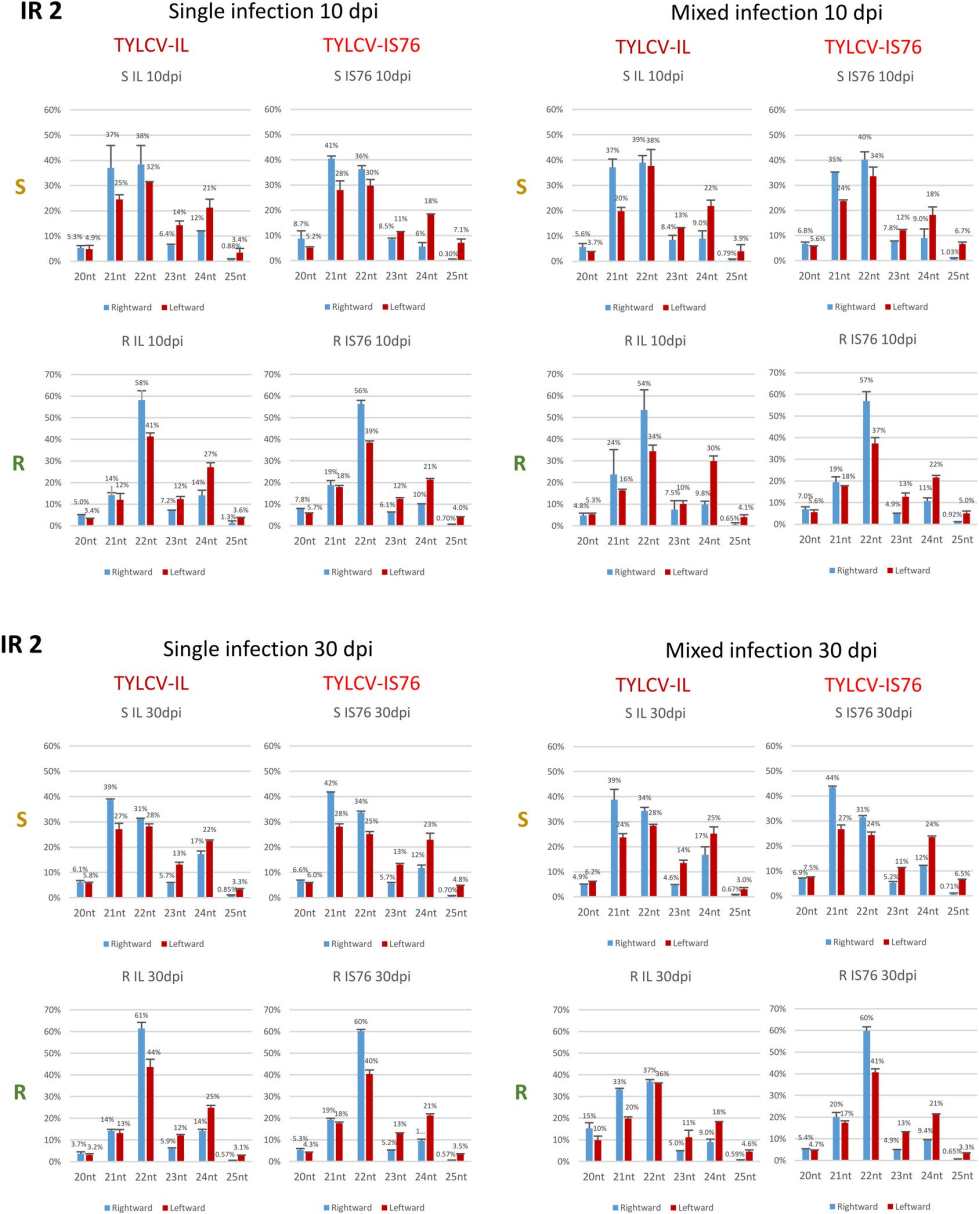
Size profiles of viral sRNAs derived from the leftward promoter-containing intergenic region 2 (IR2) in susceptible (S) and *Ty-1* resistant (R) tomato plants infected with TYLCV-IL, its recombinant derivative TYLCV-IS76 or a combination thereof (IL+S76) at 10 and 30 days post inoculation (dpi). Illumina sRNA-seq reads in the size range from 20 to 25 nts mapped to the viral genome the virion (rightward) and complementary (leftward) strands of the IR1 were counted and percentages (%) of 6 individual size-classes in the total 20–25 nt viral reads (set to 100%) were calculated and plotted as bar graphs, with blue and red bars representing rightward and leftward strands, respectively. In all panels, the percentages are for two biological replicates per each condition with the standard error shown with a capped vertical line and the mean value indicated above.

In the *A*. *thaliana*-bipartite begomovirus system, the biogenesis of 21, 22 and 24 nt viral siRNAs is mediated by DCL4, DCL2 and DCL3, respectively, which all contribute to post-transcriptional and transcriptional silencing of viral genes [30,31]. Thus, in addition to boosting the overall production of viral siRNAs by all three tomato DCLs targeting both IL and IS76, RDRγ more pronouncedly enhances the activities of DCL2 and DCL3. As argued above, RDRγ might function indirectly by promoting bidirectional readthrough transcription of the entire virus genome or directly by converting viral ssRNA transcripts into dsRNAs. In the absence of functional RDRγ in S plants, the hotspots of the dominant 21 nt class were restricted to the transcription units in all conditions, suggesting that DCL4 might preferentially process dsRNAs composed of mRNAs and readthrough transcripts of opposite polarity. In contrast, the hotspots of the less abundant 22 nt class and, much more pronouncedly, those of the low-abundance 24 nt class were also spread to the IR and the terminator region, especially at 30 dpi (S4 Dataset). Thus, DCL3 and DCL2 might preferentially process dsRNAs composed of readthrough transcripts of sense and antisense polarities. Indeed, both parts of the IR, where only readthrough transcripts might form dsRNAs, generated relatively higher proportions of 22 and 24 nt siRNAs (Figs 10 and 11) than the Pol II units (S4 Fig). In R plants, the proportion of 21 nt siRNAs derived from the IR was strongly reduced in favour of 22 nt siRNAs and, to a lesser extent, 24 nt siRNAs, and as a result the 22 nt class became dominant (Figs 10 and 11). This is despite the hotspots of both 22 nt and 21 nt siRNAs became more evenly distributed along both strands of the entire IL and IS76 genomes (S4 Dataset). Similar alterations in the sRNA size profile were also observed in the Pol II units, although the proportion of 21 nt siRNAs was less strongly reduced (S4 Fig). These findings suggest that, in addition to boosting bidirectional transcription that might generate dsRNA substrates for all three DCLs, RDRγ might also convert ssRNA templates into dsRNA substrates preferentially processed by DCL2 and less preferentially by DCL3.

Notably, the proportion of 24 nt siRNAs derived from all genome regions is higher in IL-derived sRNAs compared to IS76-derived sRNAs in most conditions, except for coinfected R plans at 30 dpi where the proportions of 24 nt siRNAs produced from residual IL and highly abundant IS76 are comparable (Figs 9, 10, 11 and S4). Thus, IS76 evades DCL3 activity better than IL and, at the same time, attracts other two DCLs generating 21 and 22 nt siRNAs better than IL (except nearly eliminated IL).

Collectively, IS76 is transcribed by Pol II more efficiently than IL owing to both recombination region elements and more efficient evasion of DCL3-mediated transcriptional silencing generating 24 nt siRNAs. More efficient transcription of IS76 facilitates replication of its DNA and accelerates transition from replication to encapsidation, but at the same time attracts DCL2 and DCL4 that mediate post-transcriptional silencing. These properties of IS76 explain its selective advantage and competitiveness in mixed infections with IL, both in the absence and presence of functional RDRγ.

Interestingly, the higher abundance of complementary strand-derived sRNAs observed within the IR and the V2-V1 unit in R plants is largely due to increased proportion of the 24 nt siRNAs that exhibit the complementary strand bias in both R and S plants in most conditions (S4 Dataset). Strand biases in viral sRNA profiles along the viral genome likely result from differential sequence-specific stability of sRNAs produced by DCLs in a form of duplexes from longer dsRNA precursors and then sorted by AGO proteins. AGOs form stable complexes with guide strands of the sRNA duplexes, and discard their passenger strands, leading to degradation of the latter. As the guide strand is selected by AGOs based on the size, 5´-nucleotide identity and other sequence features [29], differences in size and nucleotide composition of viral siRNA duplexes processed by DCLs from different types of dsRNA precursors might result in local hotspots and strand biases.

Comparison of 5’-terminal nucleotide identities of viral sRNAs did not reveal any substantial difference between IL and IS76 (S2D Dataset). In both viruses, 21 and 22 nt siRNAs predominantly possess 5’U (60–70%) followed by 5’A (~20%), suggesting their preferential association with AGO1 (5’U) and less preferential with AGO2 (5’A), whereas 24 nt siRNAs possess predominantly 5’A and 5’U (~40–50% each), suggesting their preferential association with AGO4 clade proteins (5’A) and an as-yet unknown AGO (5’U). The sRNA 5’-nucleotide profiles of IL and IS76 were similar between S and R plants at both time-points, indicating that RDRγ does not influence the sorting of viral sRNAs by AGOs.

### *Ty-1* gene is constitutively overexpressed in R plants

To complement the viral siRNA profiling results, we analysed our mRNA-seq data for expression levels of *Ty-1* and other tomato genes implicated in siRNA biogenesis and function.

**Fig 12:**
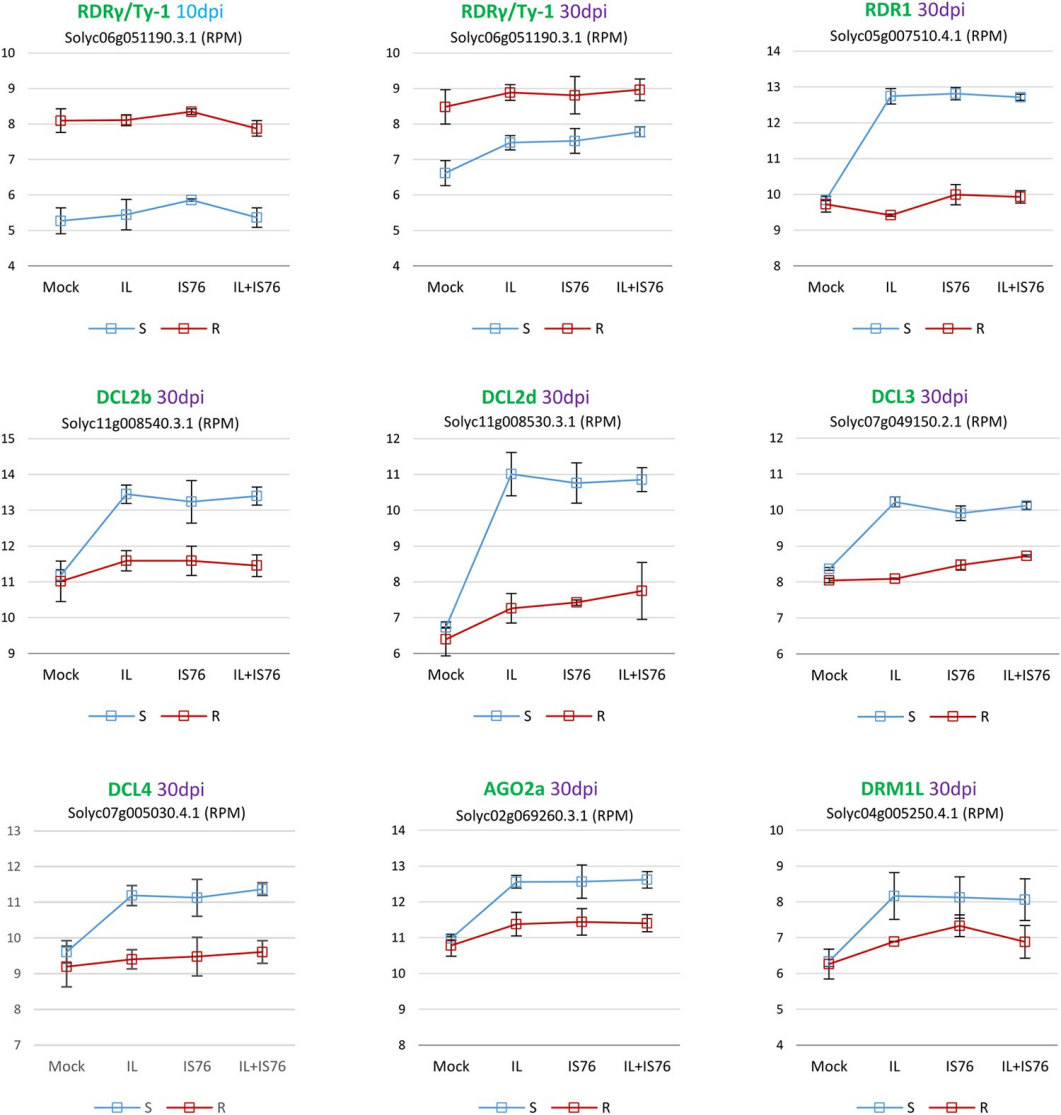
Silencing-related tomato genes differentially expressed in susceptible (S) and *Ty-1* resistant (R) tomato plants mock-inoculated vs infected with TYLCV-IL, its recombinant derivative TYLCV-IS76 or a combination thereof (IL+S76) at 10 or 30 days post inoculation (dpi). Charts plot the counts of Illumina mRNA-seq reads representing mRNAs of the RNA-dependent RNA polymerase (RDR) family genes RDRγ (Ty-1) and RDR1, the Dicer like (DCL) family genes DCL2b, DCL2d, DCL3 and DCL4, the Argonaute (AGO) family gene AGO2a and the Domain Rearranged Methyltransferase (DRM) family gene DRM1L in reads per million (RPM) of total mRNA-seq reads. The counts are for two biological replicates per each condition, with the standard error shown with a capped vertical line and the unfilled boxes positioned at the mean value levels and connected with solid lines (blue for S plants and red for R plants). The gene accession numbers (according to the annotated tomato reference genome ITAG4.1 available on Sol Genomics Network www.solgenomics.net) are given below the gene names.

Consistent with previous findings for *Ty-1* gene of the *Ty-1/ty-1* hybrid Tygress [27], this resistance gene was expressed at higher levels in our R plants (*Ty-1/ty-1* hybrid Pristyla), compared to S plants, in both mock-inoculated and virus-infected plants at both 10 and 30 dpi (Fig 12). No effect of viral infection on *Ty-1* expression levels in R plants was observed. As those levels were comparable between 10 and 30 dpi, *Ty-1* overexpression in R plants appears to be constitutive and sufficient to confer virus resistance. In contrast to R plants, expression of this gene was elevated between 10 and 30 dpi in mock-inoculated S plants and was further upregulated by both IL and IS76 as well as mixed infection, compared to mock control at 30 dpi. Notably, in both single and mixed infections of S plants, this gene was upregulated to similar levels and these levels did not reach the levels of *Ty-1* overexpression in R plants (Fig 12). At 10 dpi, expression of this gene in S plants was not altered by viral infection. As discussed previously by Verlaan et al. [27], it is not clear if the resistance conferred by the *Ty-1* allele is due to a higher transcriptional level as comparted to that of *ty-1* alleles, or the difference in amino acid sequence of the *Ty-1* allele-encoded protein. Nonetheless, *ty-1* upregulation by TYLCV-IL observed in cv. Moneymaker (*ty-1/ty-1*) by Verlaan et al. [27] and confirmed here for our susceptible *ty-1/ty-1* cultivar, nearly isogenic to the resistant *Ty-1*/*ty-1* hybrid Pristyla, would imply that RDRγ variants encoded by *ty-1* alleles could potentially contribute to antiviral defence at later stages of viral infection when their expression is upregulated. However, this contribution is not sufficient to confer resistance to TYLCV, either due to weaker functionality or insufficient accumulation of the protein even after upregulation of *ty-1* allele expression.

### IL and IS76 upregulate to similar levels the tomato genes implicated in siRNA biogenesis and function

*RDR1* gene encoding an α-clade RDR, known to be induced by RNA virus and viroid infections or salicylic acid treatment of tomato plants (reviewed in [33]), was found to be upregulated to similar levels in IL-, IS76- and [IL+IS76]-infected S plants but not in R plants (Fig 12). Tomato RDR1 can synthesize complementary RNA on ssRNA and ssDNA substrates *in vitro* [35] and its homolog in *Arabidopsis thaliana* is implicated in biogenesis of RNA virus-derived and endogenous siRNAs [46–48]. Thus, *RDR1* upregulation may contribute to the antiviral defense in S plants, but the disease caused by tomato begomoviruses indicates that such defense is evaded and particularly by IL and IS76. However, the fact that *RDR1* gene expression was not altered in *Ty-1* resistant plants (Fig 12), along with the above-mentioned findings in begomovirus-infected *Arabidopsis* wild-type vs *RDR1/RDR2/RDR6* triple mutant plants, where no substantial difference in loads or size, polarity and hotspot profiles of viral siRNAs were observed [31], would suggest a minor contribution of RDR1 (and perhaps other α-clade RDRs) to defense against TYLCV, compared to RDRγ whose anti-TYLCV activity is only partially evaded by IS76.

Among four types of tomato DCLs (DCL1 to DCL4) involved in sRNA biogenesis, two of the four genes encoding DCL2 variants (DCL2b and DCL2d) and single genes encoding DCL3 and DCL4 were found to be upregulated to similar levels in S plants infected with IL, IS76 or both viruses, compared to mock control at 30 dpi (Fig 12). *DCL2b*, being upregulated in virus-infected S plants, showed unaltered expression in R plants. This tomato gene is known to mediate both biogenesis of 22 nt endogenous sRNAs and defence against RNA virus infection [49]. *DCL2d*, being upregulated most pronouncedly in S plants, was also upregulated in R plants at 30 dpi, albeit much less pronouncedly. Thus, both variants of DCL2 might contribute to the biogenesis of 22 nt viral siRNAs and anti-TYLCV defense in S plants. The fact that production rates of 22 nt siRNAs from both IL and IS76 were boosted in R plants, compared to S plants (Fig 9), suggests that lower expression levels of the *DCL2* variants in R plants are sufficient for increased production of 22 nt siRNAs when RDRγ is boosting production of dsRNA substrates for DCLs.

*DCL4* and *DCL3*, both showing the expression profiles similar to that of *DCL2b* (Fig 12), are known to mediate the biogenesis of respectively 21 nt [50] and 24 nt [51] endogenous sRNAs in tomato plants. These DCL genes likely mediate the biogenesis of TYLCV-derived 21 and 24 nt siRNAs, respectively, as shown for their homologues in *A*. *thaliana* infected with a bipartite begomovirus [30,31]. As argued above for DCL2, expression levels of DCL4 and DCL3 appear to be sufficient for robust siRNA biogenesis in R plants. It can also be suggested that upregulation of these and other silencing-related genes depends on viral loads, which are much higher in S plants than in R plants at 30 dpi. Consistent with this hypothesis, no upregulation of silencing-related genes was observed in virus-infected S plants at 10 dpi.

Among AGO family genes, only *AGO2a* was upregulated upon late virus infection (30 dpi) in S plants and to a lesser extent in R plants (Fig 12). IL, IS76 and their combination upregulated *AGO2a* expression to similar levels. *AGO2a* is known to be co-upregulated together with *AGO1a*, *DCL2b* and *DCL2d* upon RNA virus infection in tomato [52] and to confer defense against RNA viruses in *N*. *benthamiana* [52,53]. Consistent with upregulation of *AGO2a* gene expression by IL and IS76 at 30 dpi the proportion of AGO2-associated 5’A-sRNAs of the 21 and 22 nt classes derived from both viruses were increased between 10 dpi (15–21%) and 30 dpi (18–26%) on the expense of AGO1-associated 5’U-sRNAs of these size classes (65–73% vs 56–69%) (S2D Dataset).

Finally, *DRM1L –*one of the tomato paralogs of *Domain-Rearranged Methyltransferase 2* (*DRM2*) that mediates 24 nt siRNA-directed DNA methylation in *A*. *thaliana* [28]–was upregulated upon virus infection in S plants and to a lesser extent in R plants (Fig 12). Like in the case of *AGO2a* and other silencing related genes, IL, IS76 or their combination up-regulated *DRM1L* expression to comparable levels. A role of *DRM1L* in cytosine methylation of plant and viral DNA in tomato remains to be investigated.

### Concluding remarks

In this study, we began to elucidate the molecular mechanisms underlying the *Ty-1* resistance-breaking phenotype and selective advantage of the recombinant virus IS76 as well as the strong negative impact of IS76 on its major parent TYLCV-IL in the *Ty-1* plants expressing RDRγ, an RNA-dependent RNA polymerase of the γ-clade, whose function in antiviral RNAi is poorly understood. Compared to previous studies of TYLCV-IL in susceptible (S) tomato plants using transcriptomics and sRNAomics [37] and in *Ty-1* resistant (R) vs S plants using only sRNAomics [38], we used both transcriptomics and sRNAomics for a comprehensive comparative study of TYLCL-IL, its recombinant derivative IS76 and combination thereof in both S and R tomato plants at early and late stages of infection. We found that, independent of virus identity, constitutive overexpression of RDRγ in R plants boosts the production rates of all three functional classes of viral siRNAs (21, 22 and 24 nt) from both strands of the entire virus genome and modulates DCL activities in favour of the 22 and 24 nt classes. Based on our in-depth analysis of sRNA and mRNA sequencing data for S and R plants, this is likely achieved by indirect and direct activities of RDRγ. In our current model, RDRγ might indirectly interfere with processing and polyadenylation of viral mRNAs, which would enhance readthrough transcription of circular dsDNA far beyond the poly(A) signals in both leftward and rightward directions. Both leftward and rightward readthrough transcription might span the IR and proceed even further, thus producing genome-length and longer transcripts. The leftward and rightward readthrough transcripts might pair to each other forming the dsRNAs preferentially processed by DCL3, or be converted by RDRγ to the dsRNAs preferentially processed by DCL2 and less preferentially by DCL3. Both in the presence and absence of functional RDRγ, readthrough transcripts might also pair to mature mRNAs of opposite polarities and the resulting dsRNAs would preferentially be processed by DCL4 and less preferentially by DCL2 and DCL3. This model for RDRγ-dependent and RDRγ-independent biogenesis of begomoviral siRNAs remains to be further validated using biochemical approaches.

Based on our comparative analysis of the production rates of viral siRNAs with those of viral mRNAs, IS76 appears to evade RDRγ activities and repressive siRNAs much better than IL. This is likely achieved by faster replication and accelerated transition to cell infection stages favouring the rightward transcription of viral silencing suppressor (V2) and CP genes. In our current model, V2 overexpression at earlier stages of cell infection might suppress transcriptional silencing of viral dsDNA and posttranscriptional silencing of viral mRNAs, while CP overexpression might facilitate encapsidation of viral ssDNA, followed by movement and reinfection of new cells. In mixed infection, more efficient replication and accelerated transition to overexpression of the rightward genes might provide the competitive advantage for IS76 observed in both S and R plants, while better evasion of repressive siRNAs might allow IS76 to keep high production rates of its mRNAs, even when RDRγ boosts the production rates of siRNAs from both viruses. In contrast, IL is less competitive in both S and R plants and might not evade the repressive activity of additional highly-abundant viral siRNAs derived from the transcription units and the IR sequences outside of the recombination region of IS76 which share 100% identity with the respective sequences of the IL genome. This outcompetes IL from co-infected R plants while IS76 reaches the accumulation levels of its DNA and siRNAs comparable to those in singly infected R plants.

It remains to be investigated how the alterations in the recombination region of IS76, which include 19 SNPs and 3 indels of 2, 3 and 9 nucleotides, might facilitate its replication and accelerate the transition from leftward to rightward transcription. These alterations surround the CAAT box of the core promoter driving rightward transcription and might also affect other cis-elements required for basal activity of this promoter and its transactivation by the viral protein TrAP/C2 [45]. Interestingly, a TATA-associated composite element (TACE) conserved in many genera of *Geminiviridae*, which often contains a TrAP-responsive conserved late element (CLE) or its variants with GC-rich sequences [45], is not affected by IS76 recombination, whereas an additional CLE located at the upstream position of IL is mutated in IS76 (S5 Dataset). Our results indicate that the mutation of the upstream CLE motif does not affect and even enhances the rightward promoter activity of IS76, suggesting that the TACE itself functions as a TrAP-responsive element in both TYLCV-IL and TYLCV-IS76. As CLE was proposed to bind an as-yet unidentified host transcriptional repressor protein, while TrAP interaction with this protein would de-repress the promoter activity [45], the removal of one of the two CLEs present in IL through IS76 recombination might facilitate TrAP-mediated de-repression of the rightward promoter.

The IR-based cis-elements regulating both replication efficiency and rightward-to-leftward transcription ratio might also be affected by cytosine methylation potentially directed by 24 nt viral siRNAs. In fact, a total number of cytosines on both strands of the recombination region is higher in IL (S5 Dataset). It remains to be investigated if those cytosines present in the recombination region of IL (but absent in IS76) are indeed methylated in a substantial fraction of viral circular dsDNA, thereby interfering with its transcription or replication, and whether or not RDRγ promotes cytosine methylation of viral dsDNA by boosting production of 24 nt siRNAs. Previously, cytosine methylation of TYLCV-IL DNA in susceptible tomato plants lacking the functional RDRγ was studied using bisulfite sequencing and the results revealed substantial methylation at CG, CHG and CHH sites within the entire IR as well as the V2 ORF and two parts of the C1 ORF flanking the C4 ORF, although no correlation was found between the methylation hotspots and the sRNA hotspots profiles [37]. It should be noted that the bisulfite sequencing approach used by Piedra-Aguilera et al. [37] could not distinguish between circular and linear forms of viral dsDNA. Circular dsDNA is used not only for Pol II transcription of viral mRNAs and rolling circle replication producing multiple copies of circular ssDNA, but also for recombination-dependent replication generating linear dsDNA of heterogeneous length [1]. These linear dsDNA molecules including concatemers with more-than-one copies of the viral genome might be transcribed by Pol II in both directions to produce dsRNA precursors of viral siRNAs and might also be targeted by 24 nt viral siRNAs for cytosine methylation as proposed earlier [32]. Thus, linear viral dsDNA would serve as a decoy diverting the RNAi machinery from actively transcribed circular dsDNA generating viral mRNAs. The proportion of heterogeneous linear dsDNA in total viral DNA we measured by qPCR might vary for both viruses depending on the time-point of infection or co-infection and the presence of functional RDRγ, which may contribute to the observed discrepancy between the total viral DNA loads and the viral siRNA loads (and hence their production rates estimated here).

## Material and methods

### Plant material

*Solanum lycopersicum* cultivar “Pristyla” carrying the *Ty-1* resistance gene in a heterozygous state (*Ty-1*/*ty-1*) (Gautier Semences, France) and a nearly isogenic susceptible cultivar (*ty-1*/*ty-1*) [42] were used. Seeds were sown in a nursery pot and young seedlings were transplanted in individual pots and placed in a S3 containment growth chamber with 14 h light at 26°C and 10 h dark at 24°C. They were watered with a solution containing 15:10:30 NPK fertilizer and oligoelements.

### Viral infectious clones

Two agroinfectious clones for TYLCV-IS76 [MA:SouG8:10] (GenBank accession number LN812978) and TYLCV-IL [RE:STG4:04] (GenBank accession number AM409201) were previously constructed using the binary vector pCAMBIA2300 and mobilized to the *Agrobacterium tumefaciens* strain C58 MP90 [41,42].

### Agroinoculation and sampling

Agroinfiltration or co-agroinfiltration of 14-day old seedlings with agrobacteria preparations were performed as described in Belabess et al. [42]. Two groups of tomato plants were agroinfected, one for sampling at 10 days post inoculation (dpi) and another one for sampling at 30 dpi.

The following plants were inoculated for sampling at 10 dpi. For susceptible plants, 14 seedlings were agroinoculated with TYLCV-IL, 14 with TYLCV-IS76 and 16 were co-agroinoculated with TYLCV-IL and TYLCV-IS76. For *Ty-1* resistant plants, 13 seedlings were agroinoculated with TYLCV-IL, 15 with TYLCV-IS76 and 18 were co-agroinoculated with TYLCV-IL and TYLCV-IS76.

The following plants were inoculated for sampling at 30 dpi. For susceptible plants, 8 seedlings were agroinoculated with TYLCV-IL, 8 with TYLCV-IS76 and 12 were co-agroinoculated with TYLCV-IL and TYLCV-IS76. For *Ty-1* resistant plants, 7 seedlings were agroinoculated with TYLCV-IL, 8 with TYLCV-IS76 and 11 were co-agroinoculated with TYLCV-IL and TYLCV-IS76.

As negative controls, 3 seedlings of each cultivar and for each sampling date were mock-inoculated with a preparation of agrobacteria containing an empty pCAMBIA2300 plasmid.

At 10 and 30 dpi, youngest leaves were cut from the apex of each plant and immediately frozen in dry ice before storage at -80°C. The collected leaves were pooled in two biological replicates for each condition, based on quantitative (q)PCR analysis of viral DNA loads (see below).

For the 10 and 30 dpi sampling groups, the infection status of each plant was preliminary assessed at 18 and 30 dpi, respectively, by qPCR analysis of a pool of five 4-mm diameter leaf discs collected from the youngest leaf for which five leaflets were visible (one disc per leaflet). Total DNA from the leaf disc samples was extracted using the Dellaporta protocol [54] modified as follows. Leaf tissue was ground in 400 μL extraction buffer (100 mM Tris-HCl pH 8.0, 50 mM EDTA, 500 mM NaCl, 1% SDS, 0.5 mM Na_2_SO_3_, and 0.1 mg/ml RNase A), incubated at 65°C for 10 min and centrifuged (16,000 g 10 min). One volume of cold isopropanol was added to 300 μL of the supernatant and nucleic acids were precipitated by centrifugation (16,000 g, 20 min); the pellet was washed with 70% ethanol and then resuspended in 250 μL sterile bidistilled water and stored at -20°C.

### Quantification of viral DNA loads

The load of viral DNA in each sample was measured by real-time qPCR. Each qPCR reaction was performed in a volume of 10 μL containing 2 μL of total DNA diluted 1:20, the LightCycler 480 SYBR Green I qPCR master mix (Roche, Germany), and primers. The primers for quantification of TYLCV-IL and TYLCV-IS76 [42] were added at concentrations 800 nM and 300 nM, respectively. The primers for the house-keeping tomato 25S rRNA gene [42], used as internal control for normalization of virus quantification with respect to plant DNA, were added at a concentration of 300 nM. Two technical repeats were performed for each DNA sample. The qPCR reactions were run in 384-well plates using the LightCycler 480 (Roche, Germany) with the following cycling conditions: 95°C for 10 min followed by 40 cycles each consisting of a denaturation step at 95°C for 10 sec, a hybridization step at 63°C for 40 sec for TYLCV-IL or for 20 sec for TYLCV-IS76, and an elongation step at 72°C for 15 sec. The qPCR results were analysed with the LinReg computer program [55], which calculates the initial concentration N0 for each sample, expressed in fluorescence units. This N0 value was normalized by the plant DNA concentration (N0 25S) and the amplicon size and then multiplied by 100.

### Choice of leaf samples for pooling

The plant leaf samples collected at 10 and 30 dpi were pooled according to the viral load in each plant estimated by qPCR analysis of the leaf discs collected at 18 dpi and 30 dpi, respectively, and processed as described above. For each condition, two pools of the leaf samples with similar viral loads were assembled. For single infection, the plants with the most similar and representative (close to mean) viral loads were selected and homogenously divided in two batches; to do this, the samples were ranked from the sample exhibiting the lowest virus concentration to the one with the highest concentration and selected alternatively to form the two pools. In mixed infection with TYLCV-IL and TYLCV-IS76, the criteria used for homogeneity was the ratio of the viral loads between TYLCV-IS76 and TYLCV-IL. The plants with the most similar ratio were selected and divided homogenously after ranking and alternative selection as described above. Due to the contrasted weight of available leaves at 10 and 30 dpi, the leaf samples collected at 10 dpi were from 6 plants, while the leaf samples collected at 30 dpi were from 3 plants. Each leaf pool was ground in liquid nitrogen and the resulting powder was divided for DNA and RNA extraction and stored at -80°C until use.

### DNA extraction for qPCR and Southern blot hybridization

Total DNA from the pooled leaf samples was extracted using a CTAB method of Doyle and Doyle [56]. A 0.5 ml aliquot of the CTAB buffer (100 mM Tris pH 8.0, 1.4 M NaCl, 50 mM EDTA pH 8.0, 2% CTAB, and 0.2% mercaptoethanol added before use) preheated at 60°C was added to ~0.1 g leaf tissue ground in liquid nitrogen. The mixture was incubated at 60°C for 1 hr and then centrifuged for 10 min at 10,000 rpm at room temperature (RT). The supernatant was mixed with equal volume of chloroform:isoamylalcohol (24:1). The mix was shaken for 3 min and then centrifuged for 10 min at 5,000 rpm at RT. The supernatant was transferred to a new tube and 0.66 volume of cold isopropanol was added. The tubes were stored at 4°C overnight and then centrifuged at 10,000 rpm for 10 min at RT. The supernatant was discarded and 0.5 ml of washing buffer (76% ethanol, 10 mM ammonium acetate) was added. The tubes were incubated for 20 min at RT and then centrifuged at 10,000 rpm for 5 min at RT. The supernatant was discarded and the pellet was air dried. Then, 100 μl of H_2_O and 1 μl of RNase A (10 mg/ml) were added and the mixture was incubated for 1 hr at 37°C. Two volumes of H_2_O were added, and the DNA was precipitated with 0.3 volumes of 3M sodium acetate and 2.5 volumes of cold absolute ethanol, followed by incubation for 15 min at -80°C and centrifugation at 10,000 rpm for 10 min at RT. The supernatant was discarded and the pellet was air dried at RT. The pellet was resuspended in 50 μl of H_2_O and the tubes stored at -20°C.

### RNA extraction and validation for Illumina sequencing

Total RNA extraction from the pooled leaf samples was performed using a CTAB-LiCl method as described by Golyaev et al. [57]. The integrity of high and low molecular weight RNA was evaluated by electrophoresis on respectively a 1.2% agarose-formaldehyde gel, followed by EtBr staining, and a 15% polyacrylamide-urea gel, followed by blot hybridization with a plant miR160-specific probe, as described previously [58].

### Illumina sequencing and bioinformatic analysis of viral mRNAs and viral sRNAs

Illumina sequencing was performed at Fasteris (www.fasteris.com) using the same total RNA extracts for library preparations with the Illumina stranded mRNA and the Illumina TruSeq small RNA protocols.

The mRNA libraries were multiplexed and sequenced in two flowcells of NovaSeq 6000, one flowcell with the samples from the plants collected at 10 dpi and the other one with the samples from the plants collected at 30 dpi, yielding 25’921’135 to 45’871’986 and 20’213’119 to 40’363’645 100 nt paired-end reads, respectively, and Q30 = 89.36 to 91.87 and Q30 = 89.85 to 92.00, respectively.

The sRNA libraries were multiplexed and sequenced in two flowcells of NovaSeq 6000. The first flowcell with the samples from the plants collected at 10 dpi were sequenced with 50 nt paired-end reads yielding 28’814’844 to 54’420’088 reads with Q30 = 96.46 to 96.83 for the forward read used for our follow-up analysis. The second flowcell with the samples from the plants collected at 30 dpi were sequenced with 75 nt single-end reads, yielding 37’719’583 to 50’968’605 reads with Q30 = 96.86 to 97.43.

In all cases, the libraries were de-multiplexed, followed by adapter trimming with Trimmomatic. The resulting reads were mapped using Burrow-Wheeler Aligner (BWA) 0.7.12 [59] onto the reference sequences of TYLCV-IL (AM409201) and TYLCV-IS76 (LN812978) with and without mismatches. Mapped viral reads were sorted by polarity (forward, reverse) and, in the case of sRNAs, also by size (from 15 to 34 nts) and 5’-terminal nucleotide identity (5’A, 5’U, 5’G, 5’C), and then counted (S1 Dataset for mRNA counts and S2 Dataset for sRNA counts). Single-nucleotide resolution maps of viral mRNA and sRNA reads (S3 and S4 Datasets, respectively) were generated using MISIS-2 [60].

To quantify viral mRNA and viral sRNA loads for each virus (or its selected region or strand), we used the reads aligned without mismatches. The viral read counts in each library were normalized in reads per millions (RPM) of total (viral + plant) reads.

In mixed infection, the number of reads derived from each virus (or its selected region or strand) was calculated using reads aligned without mismatches at the SNP positions present along the genome of TYLCV-IS76 and TYLCV-IL. Noteworthy, we purposely used the wild type TYLCV-IS76 infectious clone but not the laboratory generated one, TYLCV-IS76’, both of which having the same competitiveness properties [42]. Unlike TYLCV-IS76’, the wild type recombinant can be distinguished from TYLCV-IL not only by 19 SNPs and three indels of 2, 3 and 9 nts in the recombination region (between the replication origin at position 1 and the recombination breakpoint at position 84 of IL or position 76 of IS76) but also by other SNPs scattered along the viral genome (17 SNPs in the V2-V1 transcription unit, 3 SNPs in the C2-C3 transcription unit, 7 SNPs in the C1-C4 transcription unit, 2 SNPs in the intergenic region upstream of the replication origin and 1 SNP in the intergenic region downstream the recombination breakpoint (S5 Dataset). Thus, the number of reads derived from each virus (or its selected region or strand) was counted at each SNP using MISIS-2 [60] and a percentage of reads derived from each virus (or its selected region) was calculated. The average percentage at all SNPs of the viral genome (or its selected region) was applied on all parts of the genome (or its selected region) that contain no SNPs to estimate the number of reads derived from the entire genome of each virus (or its selected region) or each strand of the viral genome (or its selected region).

For viral mRNA, a production rate for each viral mRNA was calculated by dividing the mRNA counts in RPM by the DNA loads (measured by qPCR) and by the length of the mRNA from cap to poly(A) site.

For viral sRNAs, the production rate of sRNAs in the size range from 20 to 25 nts derived from the viral genome (or its selected region) and each strand of the viral genome (or its selected region) was calculated dividing the sRNA counts in RPM by the viral DNA load and, in the case of selected regions of the virus genome, by the length of each region.

Mean values with standard deviations of all those loads and production rates were calculated for the two biological replicates per each condition.

For profiling the expression of silencing-related genes, the mRNA-seq reads were mapped with BWA on the tomato reference genome ITAG4.1 available on Sol Genomics Network (www.solgenomics.net). Read were counted with the HTSeq count tool [61]. The data were then analyzed using DicoExpress [62]. Only the genes presenting a CPM (count per million) greater than or equal to 5 for at least half of the conditions were kept for further analysis. The read counts of the selected genes were normalized using the TMM method of the EdgeR package [63]. The differential analysis was performed by applying a negative binomial generalized linear model (GLM) with the EdgeR package. A gene was considered to be differentially expressed if the FDR (false discovery rate) was less than or equal to 0.05.

### Southern blot hybridization analysis with strand-specific probes

Samples of total DNA (0.5 μg) were resolved in a 0.8% agarose gel. The gel was stained with ethidium bromide for 15 min and photographed under UV light. Following denaturation and neutralization steps, DNA was transferred by capillary blotting to Hybond N+ membrane (GE Healthcare/Amersham) as described in the Hybond N+ manual. The transferred DNA was fixed to the membrane by using an UV-crosslinker (Stratagene). Blot hybridization was performed as described previously for small RNA analysis [64]. Briefly, the blot membrane was sequentially hybridized at 35°C overnight in UltraHyb-oligo buffer (Ambion) with short DNA oligonucleotides end-labelled with ^32^P gamma ATP by T4 polynucleotide kinase and purified through MicroSpin G-25 columns (GE Healthcare), following the manufacturers’ recommendations. The first probe (5’-ATCATTTCCACGCCCGTCTCGAAGGTTCGCCGA) hybridized to the complementary strand of both TYLCV-IL and TYLCV-IS76, while the second probe (5’-AAGTTCAGCCTTCGGCGAACCTTCGAGACGGGC) hybridized to the virion strand of both TYLCV-IL and TYLCV-IS76. The membrane was washed 3 times with 2X SCC, 0.5% SDS for 30 min at 35°C, and then exposed for 3 to 14 days to a phosphor screen, followed by scanning in a PhosphorImager (GE Healthcare). For the second hybridization the membrane was stripped with 0.5X SSC, 0.5% SDS for 30 min at 80°C and then with 0.1X SSC, 0.5% SDS for 30 min at 80°C. The four blot membranes shown in S2 Fig were hybridized and exposed simultaneously.

## Supporting information

S1 Text Supplementary Methods, Results and Discussion for S2 Fig. For viral DNA methylation analysis, total plant DNA was digested with cytosine methylation-dependent enzyme McrBC (NewEngland Biolab) in a total volume of 25 μL containing 2.5 μL reaction buffer, 0.25 μL albumine, 0.25 μL GTP, 0.5 μg total DNA and 15 U McrBC. The reaction was carried at 37°C for 1 hr, followed by enzyme inactivation at 65°C for 25 min. As a positive control, 0.2 μg plasmid containing a single methylated cytosine (supplied in the NewEngland Biolab McrBC kit) was mixed with 0.4 μg total plant DNA and digested in parallel as describe above. For each sample, a second aliquot of total DNA (0.5 μg) was treated in parallel under the above conditions but without McrBC. Both McrBC-digested and undigested (buffer-incubated) total DNA samples were loaded side-by-side on the 0.8% agarose gel for Southern blot hybridization analysis. Southern blot hybridization analysis with strand-specific probes revealed that circular dsDNA of IL and IS76 is resistant to McrBC digestion in S plants at 30 dpi where this form of viral DNA is above the detection threshold for both viruses. The results obtained for R plants where circular dsDNA of IS76 (but not IL) is detectable are not conclusive, although it appears to be less resistant to McrBC. However, we cannot exclude unspecific activity of McrBC digesting non-methylated dsDNA under our conditions, because McrBC was unexpectedly able to digest viral ssDNA that is produced by rolling circle replication and is not supposed to be a substrate for cytosine methylation directed by siRNAs.
(DOCX)

S1 Fig Counts of viral mRNA reads in susceptible (S) and *Ty-1* resistant (R) tomato plants infected with TYLCV-IL, its recombinant derivative TYLCV-IS76 or a combination thereof (IL+S76) at 10 (**A**) and 30 (**B**) days post inoculation (dpi). Illumina mRNA-seq reads representing the virion (rightward) and complementary (leftward) strands of the Pol II transcription units (V2-V1, C1-C4, C2-C3) and two parts of the intergenic region (IR1 and IR2) were counted in reads per million (RPM) of total (plant + viral) mRNA reads and the resulting counts plotted as bar graphs. Blue and red bars represent the rightward and leftward reads, respectively. In all cases, the counts are for two biological replicates per each condition, with the standard error shown with a capped vertical line and the mean value indicated above.
(PDF)

S2 Fig Southern blot hybridization analysis of McrBC-treated and control non-treated DNA from susceptible (S) and Ty-1 resistant (R) plants mock-inoculated or infected with IL, IS76 or IL+IS76 at 10 and 30 days post-inoculation (dpi). Total DNA extracted from tomato plants was digested with McrBC or incubated in digestion buffer without McrBC and then separated on 1% agarose gel (4 separate gels for S and R plants at 10 and 30 dpi, respectively). As control, plasmid DNA with one methylated cytosine site was spiked into total DNA from the R plant infected with IS76 at 30 dpi and loaded on one of the 4 gels. Following electrophoresis, the gels were stained with ethidium bromide and then DNA was transferred to nylon membranes by blotting and denatured. The membranes were successively hybridized with ^32^P-labelled DNA oligonucleotide probes specific for the complementary and virion strands of viral DNA and, following each hybridization, exposed together to a phosphor screen for 1 hour to 2 weeks and scanned on a PosphorImager. Note that after the first hybridization, the membranes were stripped to remove the first probe and then hybridized with the second probe. Pictures of EtBr-stained gels of the samples from 10 dpi and 30 dpi are shown in panels (**A**) and (**C**), respectively, while the respective membrane scans are shown in panels (**B**) and (**D**). Positions of plant genomic DNA (gDNA), undigested and digested plasmid DNA, viral circular double-stranded DNA (dsDNA) and viral circular single-stranded DNA (ss) are indicated.
(PDF)

S3 Fig Counts of viral sRNAs in susceptible (S) and *Ty-1* resistant (R) tomato plants infected with TYLCV-IL, its recombinant derivative TYLCV-IS76 or a combination thereof (IL+S76) at 10 (**A**) and 30 (**B**) days post inoculation (dpi). Illumina sRNA-seq reads representing the virion (rightward) and complementary (leftward) strands of the Pol II transcription units (V2-V1, C1-C4, C2-C3) and two parts of the intergenic region (IR1 and IR2) were counted in reads per million (RPM) of total (plant + viral) sRNA reads and the resulting counts plotted as bar graphs. Blue and red bars represent the rightward and leftward reads, respectively. In all cases, the counts are for two biological replicates per each condition, with the standard error shown with a capped vertical line and the mean value indicated above.
(PDF)

S4 Fig Size profiles of viral sRNAs derived from the transcription units V2-V1 (**A**), C1-C4 (**B**) and C2-C3 (**C**) in susceptible (S) and *Ty-1* resistant (R) tomato plants infected with TYLCV-IL, its recombinant derivative TYLCV-IS76 or a combination thereof (IL+S76) at 10 and 30 days post inoculation (dpi). Illumina sRNA-seq reads in the size range from 20 to 25 nts mapped to the viral genome the virion (rightward) and complementary (leftward) strands of each transcription unit were counted and percentages (%) of 6 individual size-classes in the total 20–25 nt viral reads (set to 100%) were calculated and plotted as bar graphs, with blue and red bars representing rightward and leftward strands, respectively. In all panels, the percentages are for two biological replicates per each condition, with the standard error shown with a capped vertical line and the mean value indicated above.
(PDF)

S1 Dataset Counts of Illumina mRNA-seq reads from susceptible (S) and Ty-1 resistant (R) tomato plants mock-inoculated or infected with TYLCV-IL, its recombinant derivative TYLCV-IS76 or their combination (IL+IS76) at 10 and 30 days post inoculation (dpi). The Illumina 100 nt paired-end reads from each library (two biological replicates per condition: pool 1 and pool 2) were mapped without (**A**) or with (**B**) mismatches to the reference sequences of the *Solanum lycopersicum* genome (nuclear, chloroplast and mitochondrion) and the viral genomes (IL and IS76), sorted by polarity (forward, reverse, total) and counted. The counts of plant and viral reads mapped without mismatches were then normalized per million of total reads (RPM) in each library (**C**).
(XLSX)

S2 Dataset Counts of Illumina small RNA-seq reads from susceptible (S) and Ty-1 resistant (R) tomato plants mock-inoculated or infected with TYLCV-IL, its recombinant derivative TYLCV-IS76 or their combination (IL+IS76) at 10 and 30 days post inoculation (dpi). The Illumina 15–34 nt reads from each library (two biological replicates per condition: pool 1 and pool 2) were mapped without (**A**) or with (**B**) mismatches to the reference sequences of the *Solanum lycopersicum* genome (nuclear, chloroplast and mitochondrion) and the viral genomes (IL and IS76), sorted by size (15 nt through 34 nt) and polarity (forward, reverse, total) and then counted. The counts of plant and viral reads mapped without mismatches were then normalized per million of total reads (RPM) in each library (**C**) and were also sorted by 5’-terminal nucleotide identity (5’A, 5’C, 5’G, 5’U) and then counted in percentage of total (**D**).
(XLSX)

S3 Dataset Single nucleotide resolution maps of Illumina mRNA-seq reads representing viral transcripts from susceptible (S) and Ty-1 resistant (R) tomato plants infected with TYLCV-IL, its recombinant derivative TYLCV-IS76 or their combination (IL+IS76) at 10 and 30 days post inoculation (dpi). For each condition, Illumina 100 nt paired-end reads of the two biological replicates (1 and 2) were mapped onto the reference sequences of IL and IS76 using BWA and the resulting BAM files were analysed by MISIS-2 (Seguin et al. 2016 [60]) to generate tables of reads mapped to each reference sequence with zero mismatches. The reference sequences were extended at the 3-end by 99 nts from the 5’-terminal sequence to allow for mapping RNAs derived from the circular viral genome including the first and last nucleotide of the linear reference. In each table, the first column gives nucleotide positions of the corresponding viral genome sequence. In the next columns, the positions of 5′-terminal nucleotide of sense RNAs and 3′-terminal nucleotide of antisense RNAs along the reference sequence are indicated, and the read counts are given for each RNA mapped with zero mismatches to the forward (rightward) strand (columns fwd1 and fwd2) and the reverse (leftward) strand (columns rev1 and rev2), along with the total counts of reads mapped at the respective positions of the forward (rightward) and reverse (leftward) strands in the two replicates divided by 2 (i.e., average counts). In each table file on the right side, histograms of the average counts of rightward and leftward reads are inserted with the rightward reads colored in blue and the leftward reads colored in red. In the case of mixed infections (IL+IS76), the number of reads derived from each virus was counted at each SNP using MISIS-2 (Seguin et al. 2006 [60]) and a percentage of reads derived from each virus (or its selected region) was calculated. The average percentage at all SNPs of the viral genome (or its selected region) was applied on all parts of the genome (or its selected region) that contain no SNPs to estimate the number of reads derived from the entire genome of each virus (or its selected region) or each strand of the viral genome (or its selected region).
(XLSX)

S4 Dataset Single-nucleotide resolution maps of viral 20–25 nt small (s)RNAs in susceptible (S) and Ty-1 resistant (R) tomato plants infected with TYLCV-IL, its recombinant derivative TYLCV-IS76 or their combination (IL+IS76) at 10 and 30 days post inoculation (dpi). For each condition, Illumina 20–25 nt reads of the two biological replicates were combined and mapped onto the reference sequences of IL and IS76 using BWA and the resulting BAM files were analysed by MISIS-2 (Seguin et al. 2016 [60]) to generate tables of reads mapped to each reference sequence with zero mismatches and sorted by size and polarity. The reference sequences were extended at the 3’-end by 33 nts from the 5’-terminal sequence to allow for mapping sRNAs derived from the circular viral genome (at the junction of the first and last nucleotide of the linear reference). The counts of reads mapped to the extended sequence were then added to the 5’-sequence. In each table, the first column gives nucleotide positions of the corresponding viral genome sequence. In the next columns, the positions of 5′-terminal nucleotide of sense sRNAs and 3′-terminal nucleotide of antisense siRNAs along the reference sequence are indicated, and the read counts are given for each sRNA of 20-, 21-, 22-, 23-, 24- and 25-nt classes mapped with zero mismatches to the forward (rigthward) strand (columns 20 rightward, 21 rightward, 22 rightward, 23 rightward, 24 rightward, 25 rightward) and the reverse (leftward) strand (columns 20 leftward, 21 leftward, 22 leftward, 23 leftward, 24 leftward, 25 leftward), along with the total counts of 20–25 nt sRNAs mapped on the forward (rightward) and reverse (leftward) strands. In each table file on the right side, histograms of three major size-classes of siRNAs (21, 22, and 24 nt rightward and leftward reads) are inserted with the rightward reads colored in blue and the leftward reads colored in red. In the case of mixed infections (IL+IS76), the number of reads derived from each virus was counted at each SNP using MISIS-2 (Seguin et al. 2006 [60]) and a percentage of reads derived from each virus (or its selected region) was calculated. The average percentage at all SNPs of the viral genome (or its selected region) was applied on all parts of the genome (or its selected region) that contain no SNPs to estimate the number of reads derived from the entire genome of each virus (or its selected region) or each strand of the viral genome (or its selected region).
(XLSX)

S5 Dataset Reference sequences of the viral genome IL and IS76 and their pairwise alignment. The start and stop codons of viral ORFs are coloured in red and underlined, the CAAT and TATA-boxes of the promoters coloured in brick red, the TATA-associated composite element (TACE) and conserved late elements (CLE) highlighted in green and cyan, respectively, the iterons highlighted in grey and SNPs and indels highlighted in yellow.
(PDF)
