# Supplementary material for: Transcriptome and small RNAome profiling uncovers how a recombinant begomovirus evades RDRγ-mediated silencing of viral genes and outcompetes its parental virus in mixed infection
Source: PLoS Pathog. 2024 Jan 12;20(1):e1011941. doi: 10.1371/journal.ppat.1011941 (PMC10810479; doi:10.1371/journal.ppat.1011941)
Supplement: S1 Text — For viral DNA methylation analysis, total plant DNA was digested with cytosine methylation-dependent enzyme McrBC (NewEngland Biolab) in a total volume of 25 μL containing 2.5 μL reaction buffer, 0.25 μL albumine, 0.25 μL GTP, 0.5 μg total DNA and 15 U McrBC. The reaction was carried at 37°C for 1 hr, followed by enzyme inactivation at 65°C for 25 min. As a positive control, 0.2 μg plasmid containing a single methylated cytosine (supplied in the NewEngland Biolab McrBC kit) was mixed with 0.4 μg total plant DNA and digested in parallel as describe above. For each sample, a second aliquot of total DNA (0.5 μg) was treated in parallel under the above conditions but without McrBC. Both McrBC-digested and undigested (buffer-incubated) total DNA samples were loaded side-by-side on the 0.8% agarose gel for Southern blot hybridization analysis. Southern blot hybridization analysis with strand-specific probes revealed that circular dsDNA of IL and IS76 is resistant to McrBC digestion in S plants at 30 dpi where this form of viral DNA is above the detection threshold for both viruses. The results obtained for R plants where circular dsDNA of IS76 (but not IL) is detectable are not conclusive, although it appears to be less resistant to McrBC. However, we cannot exclude unspecific activity of McrBC digesting non-methylated dsDNA under our conditions, because McrBC was unexpectedly able to digest viral ssDNA that is produced by rolling circle replication and is not supposed to be a substrate for cytosine methylation directed by siRNAs. (DOCX) [file ppat.1011941.s001.docx]

S1 Text.

For viral DNA methylation analysis, total plant DNA was digested with cytosine methylation-dependent enzyme McrBC (NewEngland Biolab) in a total volume of 25 µL containing 2.5 µL reaction buffer, 0.25 µL albumine, 0.25 µL GTP, 0.5 µg total DNA and 15 U McrBC. The reaction was carried at 37oC for 1 hr, followed by enzyme inactivation at 65oC for 25 min. As a positive control, 0.2 µg plasmid containing a single methylated cytosine (supplied in the NewEngland Biolab McrBC kit) was mixed with 0.4 µg total plant DNA and digested in parallel as describe above. For each sample, a second aliquot of total DNA (0.5 µg) was treated in parallel under the above conditions but without McrBC. Both McrBC-digested and undigested (buffer-incubated) total DNA samples were loaded side- by-side on the 0.8 % agarose gel for Southern blot hybridization analysis.

Southern blot hybridization analysis with strand-specific probes revealed that circular dsDNA of IL and IS76 is resistant to McrBC digestion in S plants at 30 dpi where this form of viral DNA is above the detection threshold for both viruses. The results obtained for R plants where circular dsDNA of IS76 (but not IL) is detectable are not conclusive, although it appears to be less resistant to McrBC. However, we cannot exclude unspecific activity of McrBC digesting non-methylated dsDNA under our conditions, because McrBC was unexpectedly able to digest viral ssDNA that is produced by rolling circle replication and is not supposed to be a substrate for cytosine methylation directed by siRNAs.
