# Supplementary material for: Transcriptome and small RNAome profiling uncovers how a recombinant begomovirus evades RDRγ-mediated silencing of viral genes and outcompetes its parental virus in mixed infection
Source: PLoS Pathog. 2024 Jan 12;20(1):e1011941. doi: 10.1371/journal.ppat.1011941 (PMC10810479; doi:10.1371/journal.ppat.1011941)
Supplement: S1 Fig — Counts of viral mRNA reads in susceptible (S) and Ty-1 resistant (R) tomato plants infected with TYLCV-IL, its recombinant derivative TYLCV-IS76 or a combination thereof (IL+S76) at 10 (A) and 30 (B) days post inoculation (dpi). Illumina mRNA-seq reads representing the virion (rightward) and complementary (leftward) strands of the Pol II transcription units (V2-V1, C1-C4, C2-C3) and two parts of the intergenic region (IR1 and IR2) were counted in reads per million (RPM) of total (plant + viral) mRNA reads and the resulting counts plotted as bar graphs. Blue and red bars represent the rightward and leftward reads, respectively. In all cases, the counts are for two biological replicates per each condition, with the standard error shown with a capped vertical line and the mean value indicated above. (PDF) [file ppat.1011941.s002.pdf]

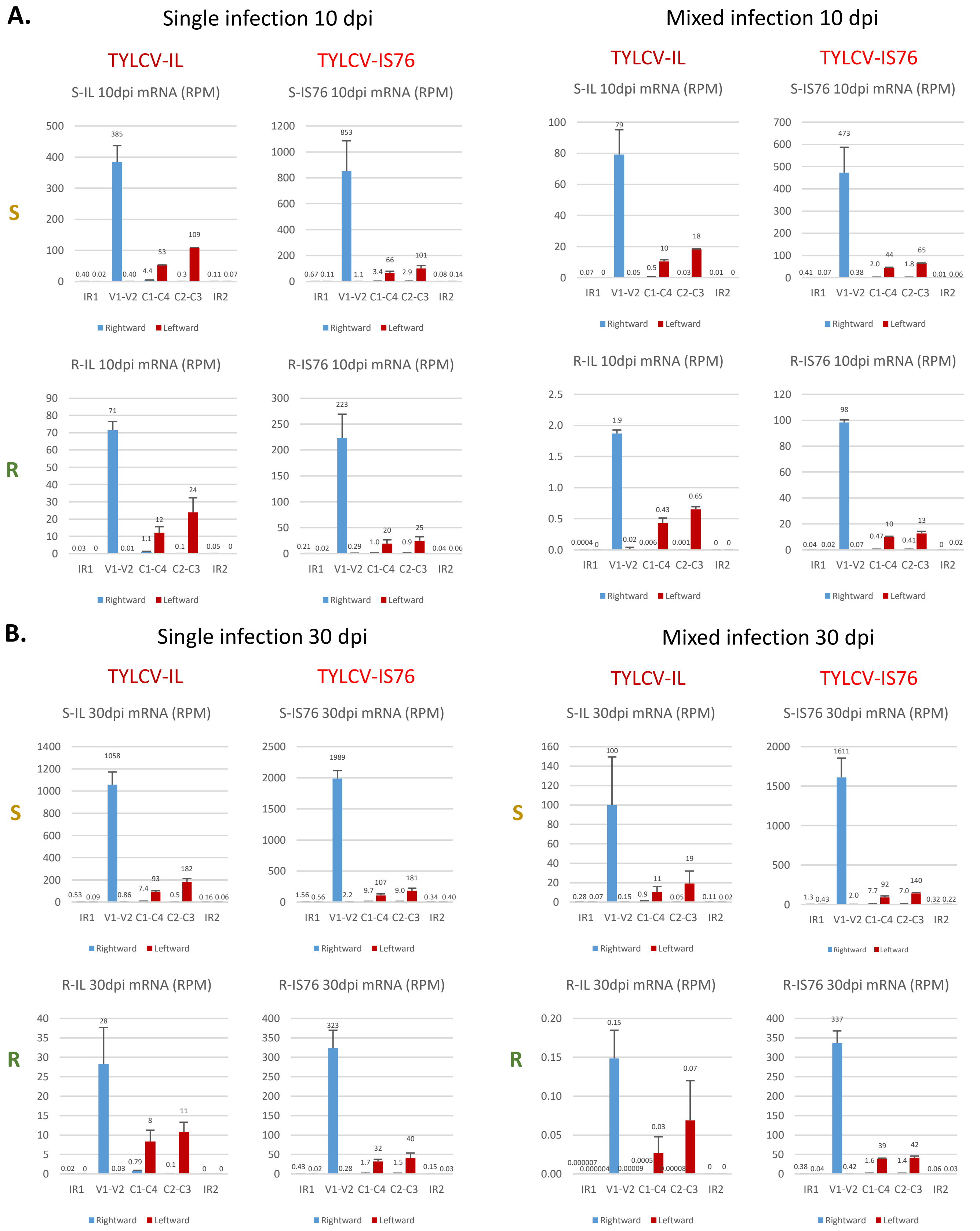

S1 Figure. Counts of viral mRNA reads in susceptible (S) and *Ty-1* resistant (R) tomato plants infected with TYLCV-IL, its recombinant derivative TYLCV-IS76 or a combination thereof (IL+S76) at 10 (**A**) and 30 (**B**) days post inoculation (dpi). Illumina mRNA-seq reads representing the virion (rightward) and complementary (leftward) strands of the Pol II transcription units (V2-V1, C1-C4, C2-C3) and two parts of the intergenic region (IR1 and IR2) were counted in reads per million (RPM) of total (plant + viral) mRNA reads and the resulting counts plotted as bar graphs. Blue and red bars represent the rightward and leftward reads, respectively. In all cases, the counts are for two biological replicates per each condition, with the standard error shown with a capped vertical line and the mean value indicated above.
