# Supplementary material for: Transcriptome and small RNAome profiling uncovers how a recombinant begomovirus evades RDRγ-mediated silencing of viral genes and outcompetes its parental virus in mixed infection
Source: PLoS Pathog. 2024 Jan 12;20(1):e1011941. doi: 10.1371/journal.ppat.1011941 (PMC10810479; doi:10.1371/journal.ppat.1011941)
Supplement: S2 Fig — Total DNA extracted from tomato plants was digested with McrBC or incubated in digestion buffer without McrBC and then separated on 1% agarose gel (4 separate gels for S and R plants at 10 and 30 dpi, respectively). As control, plasmid DNA with one methylated cytosine site was spiked into total DNA from the R plant infected with IS76 at 30 dpi and loaded on one of the 4 gels. Following electrophoresis, the gels were stained with ethidium bromide and then DNA was transferred to nylon membranes by blotting and denatured. The membranes were successively hybridized with 32P-labelled DNA oligonucleotide probes specific for the complementary and virion strands of viral DNA and, following each hybridization, exposed together to a phosphor screen for 1 hour to 2 weeks and scanned on a PosphorImager. Note that after the first hybridization, the membranes were stripped to remove the first probe and then hybridized with the second probe. Pictures of EtBr-stained gels of the samples from 10 dpi and 30 dpi are shown in panels (A) and (C), respectively, while the respective membrane scans are shown in panels (B) and (D). Positions of plant genomic DNA (gDNA), undigested and digested plasmid DNA, viral circular double-stranded DNA (dsDNA) and viral circular single-stranded DNA (ss) are indicated. (PDF) [file ppat.1011941.s003.pdf]

S2 Figure. Southern blot hybridization analysis of McrBC-treated and control non-treated DNA from susceptible (S) and *Ty-1* resistant (R) plants mock-inoculated or infected with IL, IS76 or IL+IS76 at 10 and 30 days post-inoculation (dpi). Total DNA extracted from tomato plants was digested with McrBC or incubated in digestion buffer without McrBC and then separated on 1% agarose gel (4 separate gels for S and R plants at 10 and 30 dpi, respectively). As control, plasmid DNA with one methylated cytosine site was spiked into total DNA from the R plant infected with IS76 at 30 dpi and loaded on one of the 4 gels. Following electrophoresis, the gels were stained with ethidium bromide and then DNA was transferred to nylon membranes by blotting and denatured. The membranes were successively hybridized with ^{32}P-labelled DNA oligonucleotide probes specific for the complementary and virion strands of viral DNA and, following each hybridization, exposed together to a phosphor screen for 1 hour to 2 weeks and scanned on a PosphorImager. Note that after the first hybridization, the membranes were stripped to remove the first probe and then hybridized with the second probe. Pictures of EtBr-stained gels of the samples from 10 dpi and 30 dpi are shown in panels (**A**) and (**C**), respectively, while the respective membrane scans are shown in panels (**B**) and (**D**). Positions of plant genomic DNA (gDNA), undigested and digested plasmid DNA, viral circular double-stranded DNA (dsDNA) and viral circular single-stranded DNA (ss) are indicated.

(A)

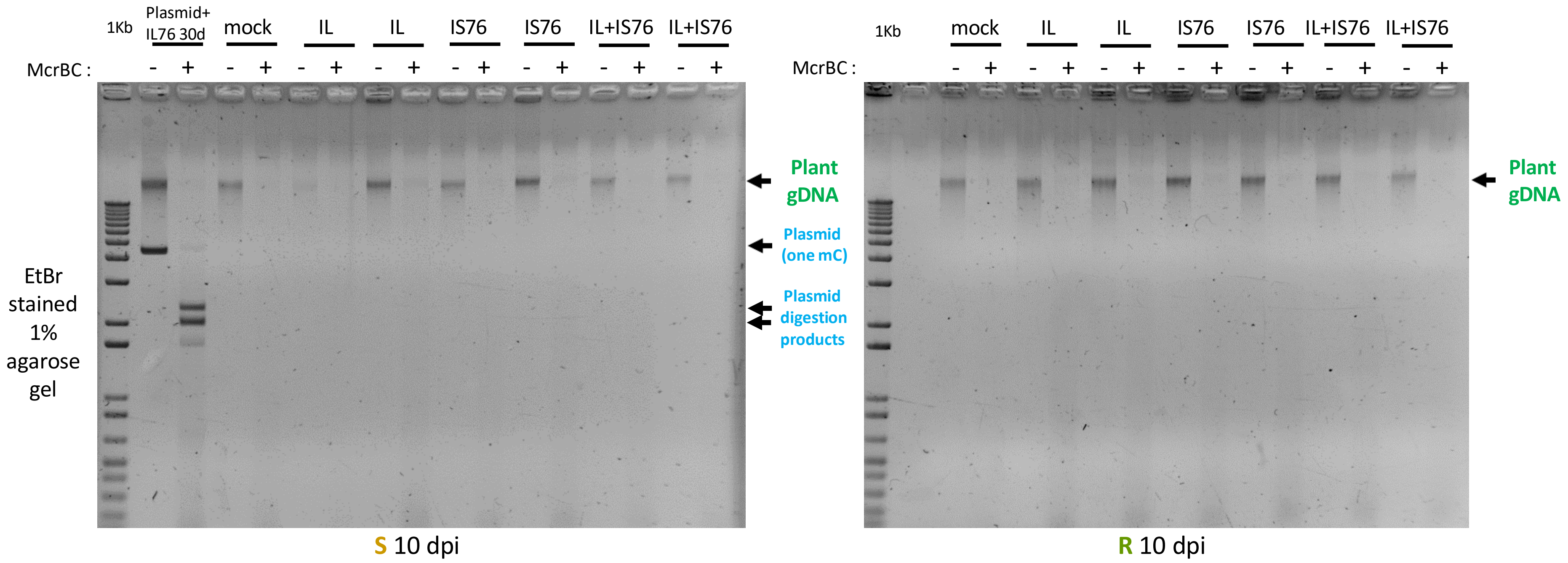
1Kb
Plasmid+ IL76 30d
mock
IL
IL
IS76
IS76
IL+IS76
IL+IS76
McrBC :
- + - + - + - + - + - + - + - +
EtBr stained 1% agarose gel
Plant gDNA
Plasmid (one mC)
Plasmid digestion products
S 10 dpi
1Kb
mock
IL
IL
IS76
IS76
IL+IS76
IL+IS76
McrBC :
- + - + - + - + - + - + - +
Plant gDNA
R 10 dpi

(B)

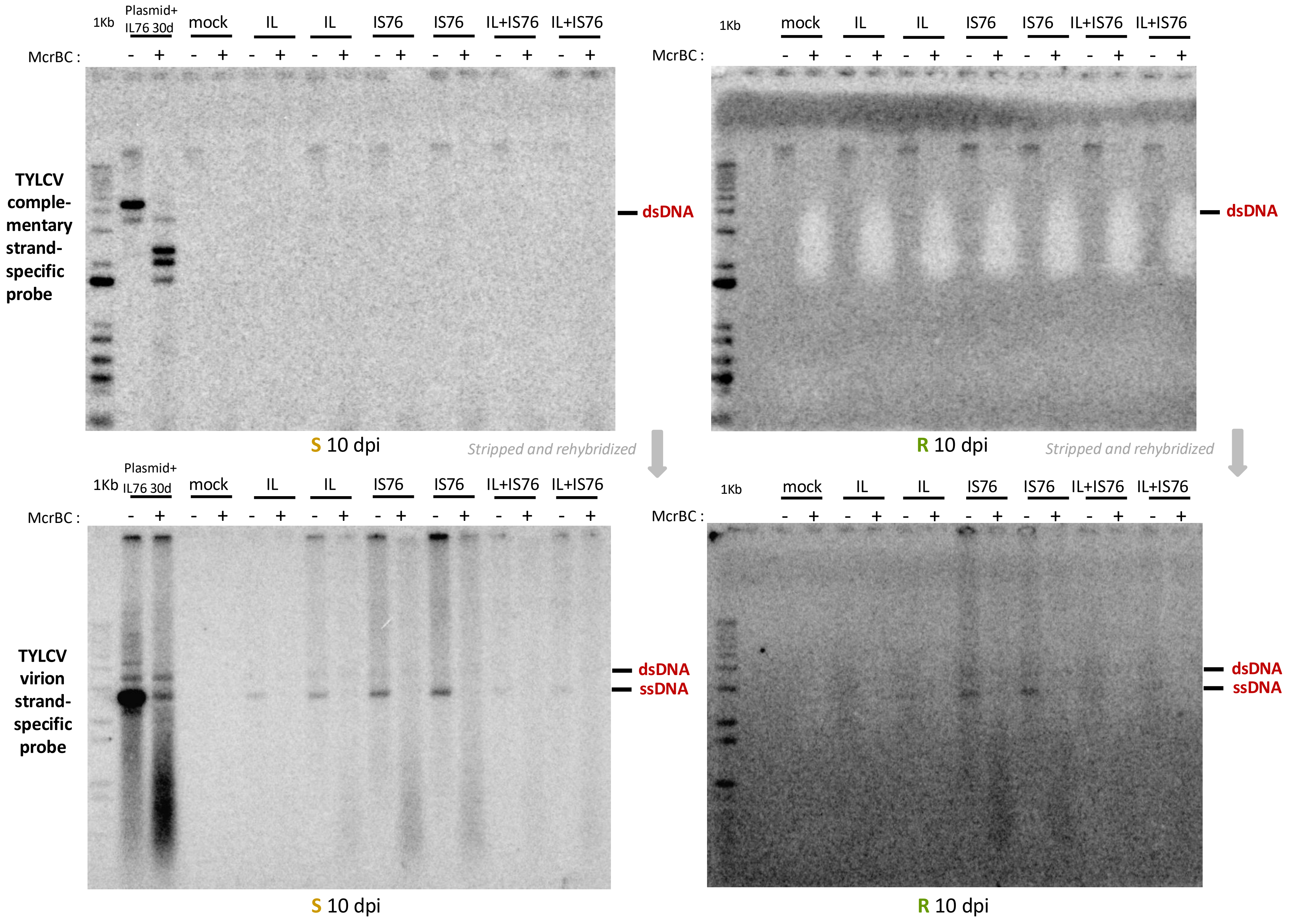
1Kb
Plasmid+ IL76 30d
mock
IL
IL
IS76
IS76
IL+IS76
IL+IS76
McrBC :
- + - + - + - + - + - + - + - +
TYLCV complementary strand-specific probe
dsDNA
S 10 dpi
Stripped and rehybridized
1Kb
mock
IL
IL
IS76
IS76
IL+IS76
IL+IS76
McrBC :
- + - + - + - + - + - + - +
dsDNA
R 10 dpi
Stripped and rehybridized
1Kb
Plasmid+ IL76 30d
mock
IL
IL
IS76
IS76
IL+IS76
IL+IS76
McrBC :
- + - + - + - + - + - + - + - +
TYLCV virion strand-specific probe
dsDNA
ssDNA
S 10 dpi
1Kb
mock
IL
IL
IS76
IS76
IL+IS76
IL+IS76
McrBC :
- + - + - + - + - + - + - +
dsDNA
ssDNA
R 10 dpi

(C)

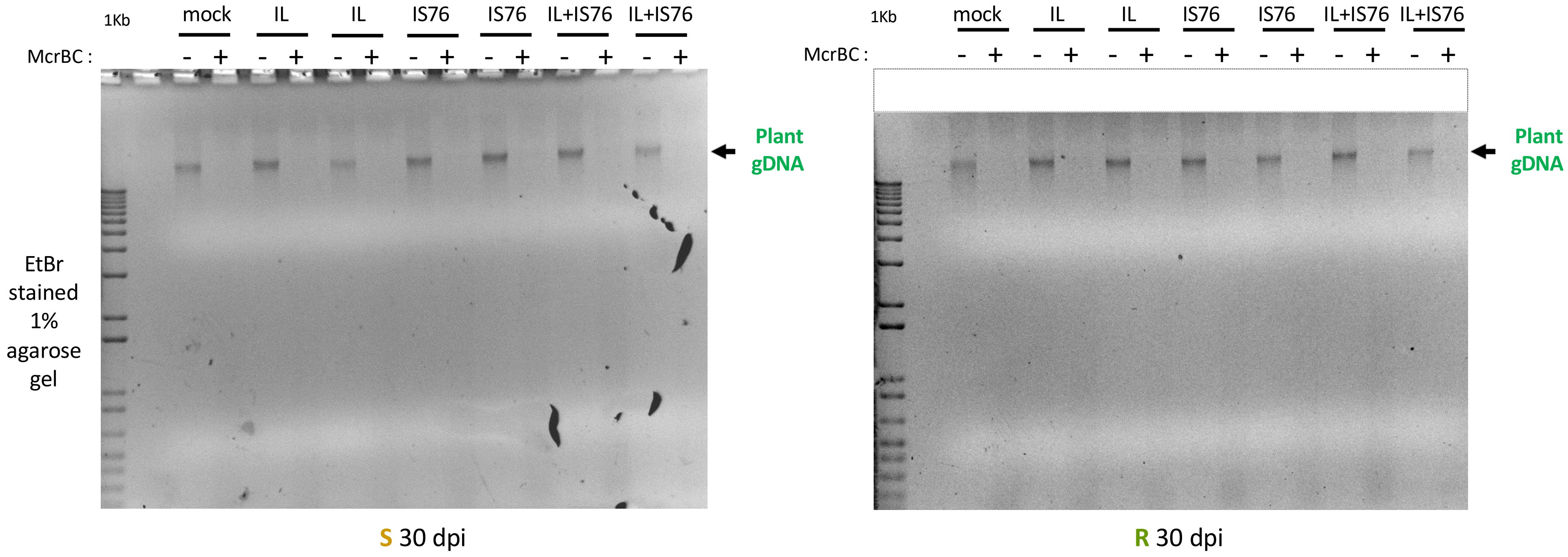
1Kb
mock
IL
IL
IS76
IS76
IL+IS76
IL+IS76
McrBC :
- + - + - + - + - + - + - +
Plant gDNA
EtBr stained 1% agarose gel
S 30 dpi
1Kb
mock
IL
IL
IS76
IS76
IL+IS76
IL+IS76
McrBC :
- + - + - + - + - + - + - +
Plant gDNA
R 30 dpi

(D)

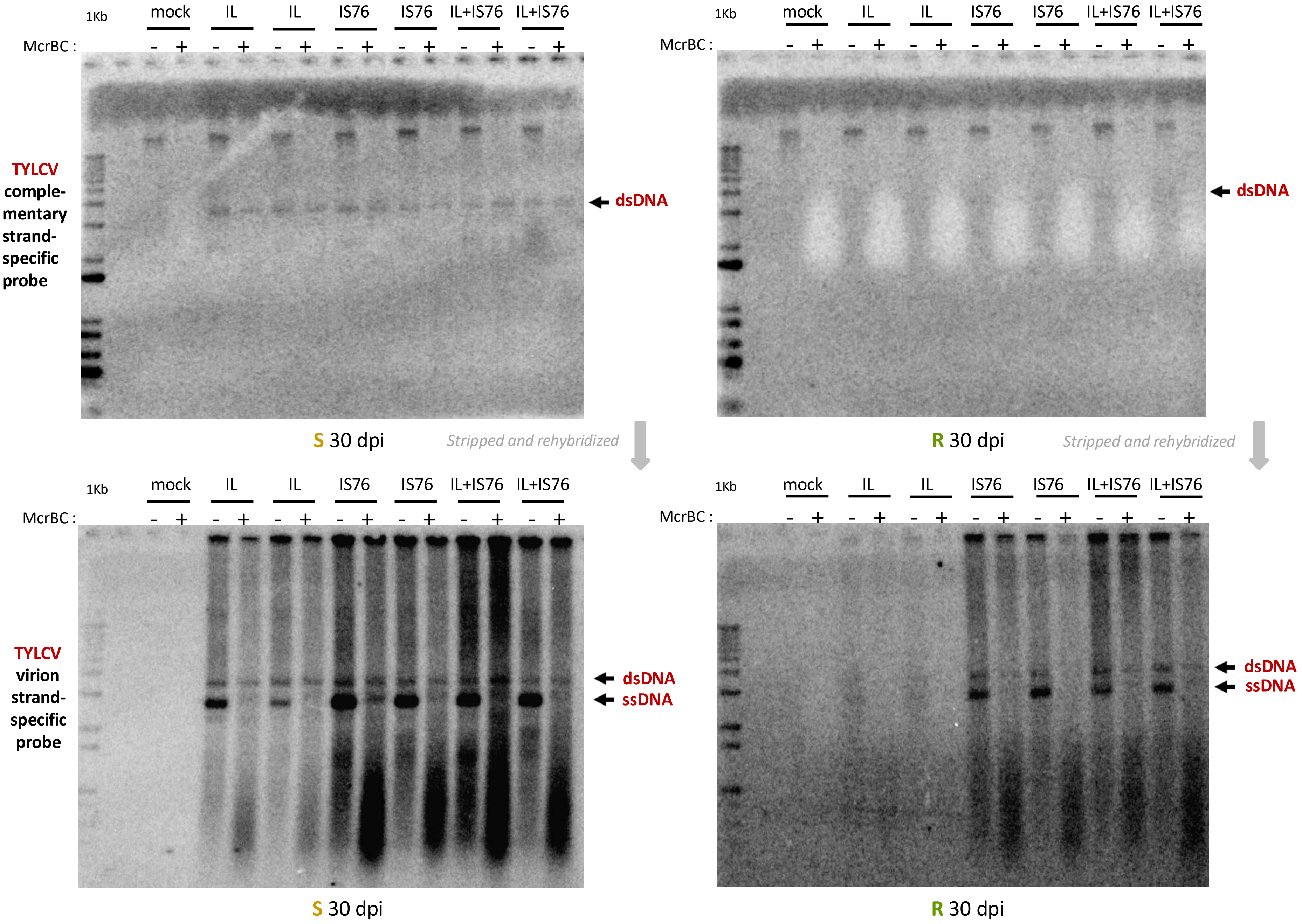
1Kb
mock
IL
IL
IS76
IS76
IL+IS76
IL+IS76
McrBC :
- + - + - + - + - + - + - +
TYLCV complementary strand-specific probe
dsDNA
S 30 dpi
Stripped and rehybridized
R 30 dpi
Stripped and rehybridized
TYLCV virion strand-specific probe
dsDNA
ssDNA
S 30 dpi
R 30 dpi
