# Supplementary material for: Transcriptome and small RNAome profiling uncovers how a recombinant begomovirus evades RDRγ-mediated silencing of viral genes and outcompetes its parental virus in mixed infection
Source: PLoS Pathog. 2024 Jan 12;20(1):e1011941. doi: 10.1371/journal.ppat.1011941 (PMC10810479; doi:10.1371/journal.ppat.1011941)
Supplement: S4 Fig — Size profiles of viral sRNAs derived from the transcription units V2-V1 (A), C1-C4 (B) and C2-C3 (C) in susceptible (S) and Ty-1 resistant (R) tomato plants infected with TYLCV-IL, its recombinant derivative TYLCV-IS76 or a combination thereof (IL+S76) at 10 and 30 days post inoculation (dpi). Illumina sRNA-seq reads in the size range from 20 to 25 nts mapped to the viral genome the virion (rightward) and complementary (leftward) strands of each transcription unit were counted and percentages (%) of 6 individual size-classes in the total 20–25 nt viral reads (set to 100%) were calculated and plotted as bar graphs, with blue and red bars representing rightward and leftward strands, respectively. In all panels, the percentages are for two biological replicates per each condition, with the standard error shown with a capped vertical line and the mean value indicated above. (PDF) [file ppat.1011941.s005.pdf]

S4 Figure. Size profiles of viral sRNAs derived from the transcription units V2-V1 (**A**), C1-C4 (**B**) and C2-C3 (**C**) in susceptible (S) and *Ty-1* resistant (R) tomato plants infected with TYLCV-IL, its recombinant derivative TYLCV-IS76 or a combination thereof (IL+S76) at 10 and 30 days post inoculation (dpi). Illumina sRNA-seq reads in the size range from 20 to 25 nts mapped to the viral genome the virion (rightward) and complementary (leftward) strands of each transcription unit were counted and percentages (%) of 6 individual size-classes in the total 20-25 nt viral reads (set to 100%) were calculated and plotted as bar graphs, with blue and red bars representing rightward and leftward strands, respectively. In all panels, the percentages are for two biological replicates per each condition, with the standard error shown with a capped vertical line and the mean value indicated above.

A.

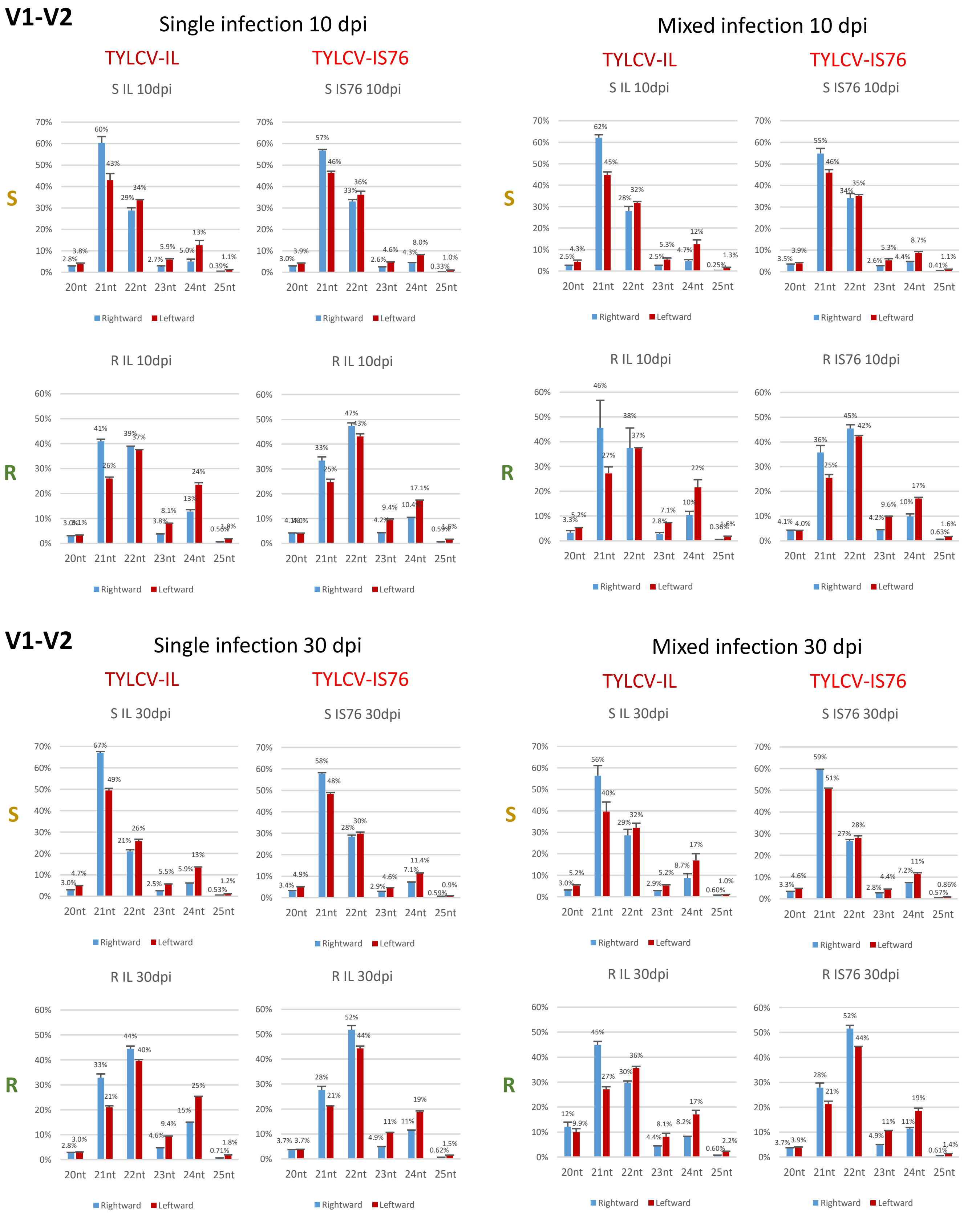

B.

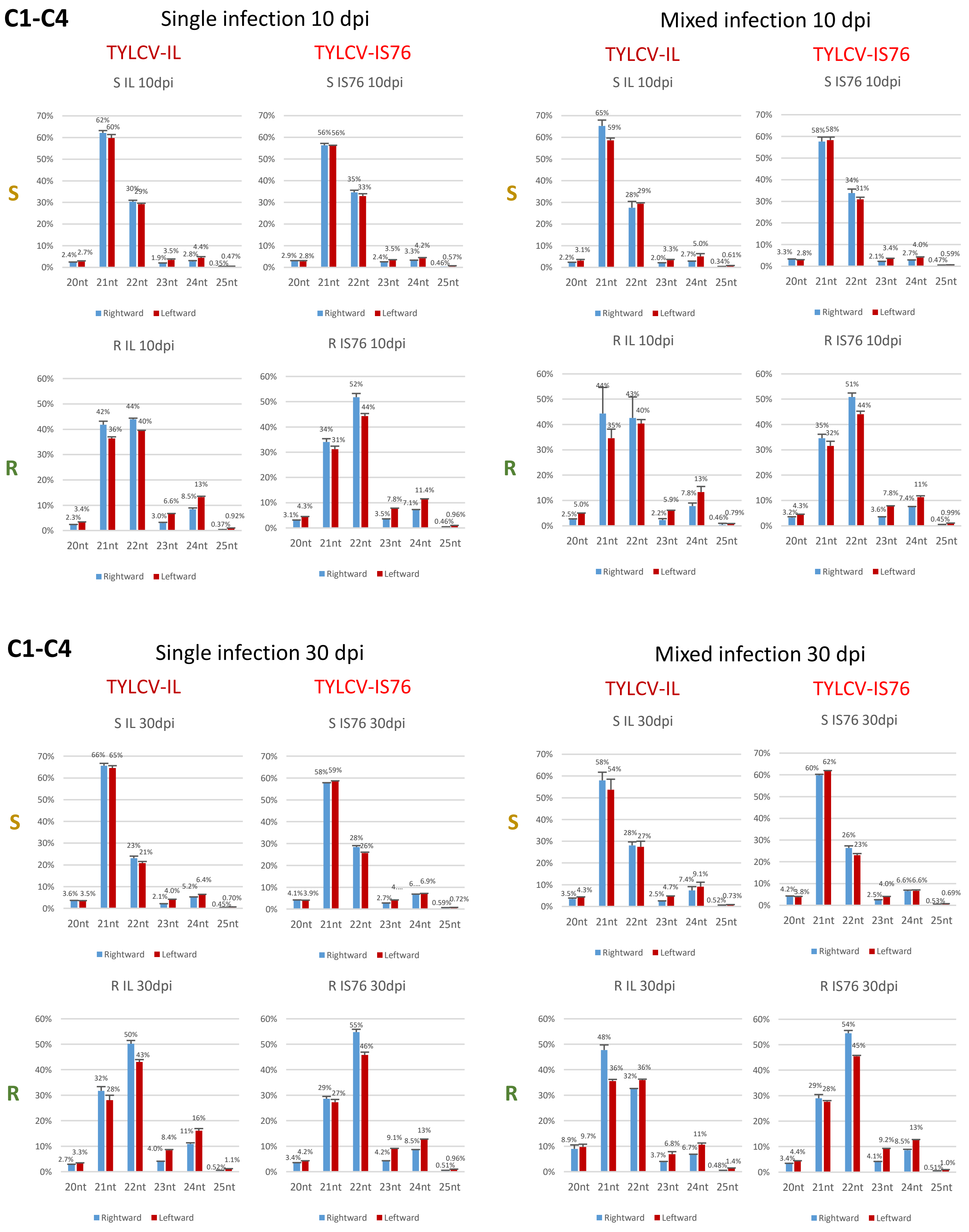
C1-C4
Single infection 10 dpi
Mixed infection 10 dpi
TYLCV-IL
TYLCV-IS76
S IL 10dpi
S IS76 10dpi
R IL 10dpi
R IS76 10dpi
S
R
C1-C4
Single infection 30 dpi
Mixed infection 30 dpi
S IL 30dpi
S IS76 30dpi
R IL 30dpi
R IS76 30dpi
0%
10%
20%
30%
40%
50%
60%
70%
20nt
21nt
22nt
23nt
24nt
25nt
Rightward
Leftward

C.

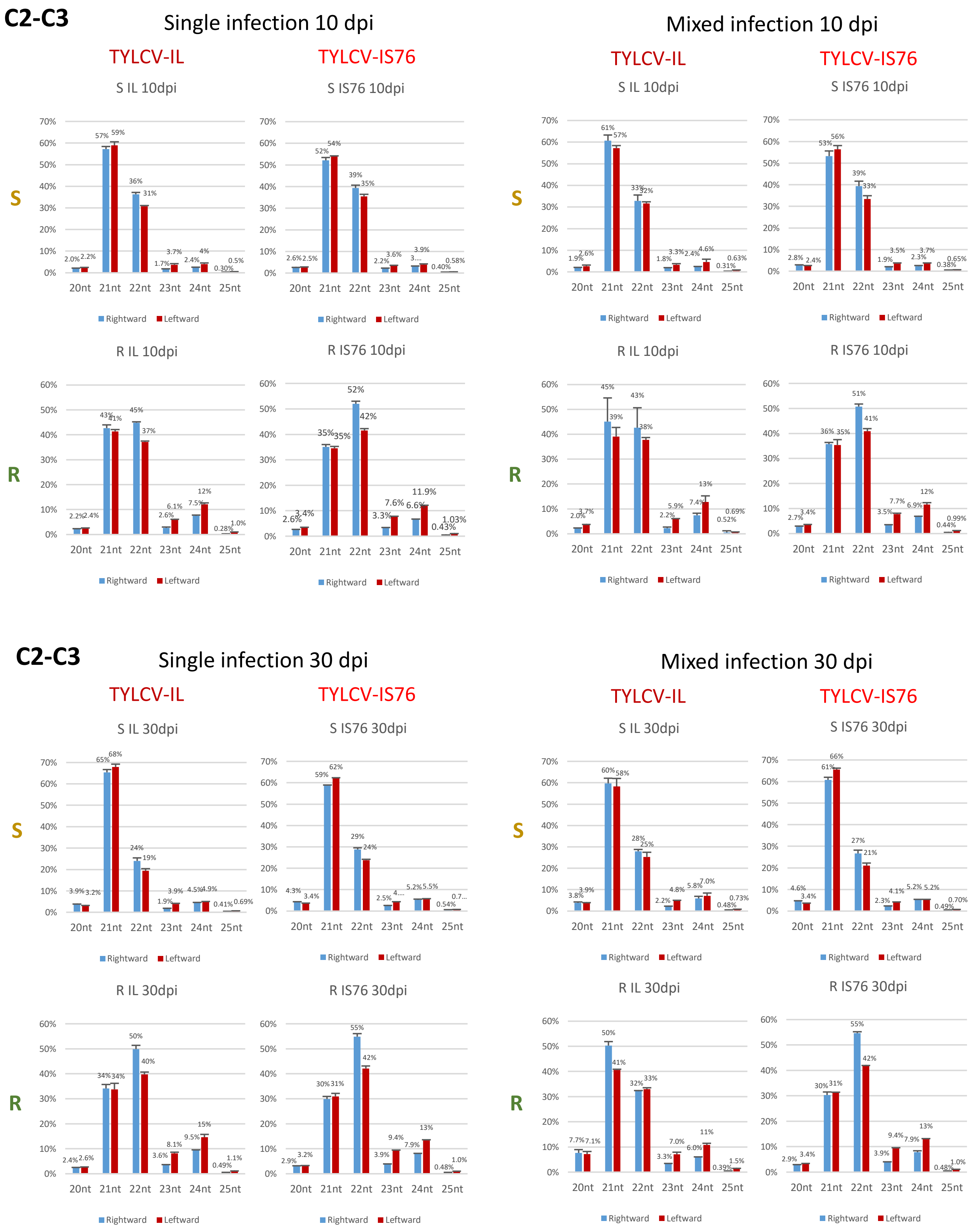
C2-C3
Single infection 10 dpi
Mixed infection 10 dpi
TYLCV-IL
TYLCV-IS76
S IL 10dpi
S IS76 10dpi
R IL 10dpi
R IS76 10dpi
C2-C3
Single infection 30 dpi
Mixed infection 30 dpi
S IL 30dpi
S IS76 30dpi
R IL 30dpi
R IS76 30dpi
S
R
20nt 21nt 22nt 23nt 24nt 25nt
Rightward
Leftward
