# Supplementary material for: Transcriptome and small RNAome profiling uncovers how a recombinant begomovirus evades RDRγ-mediated silencing of viral genes and outcompetes its parental virus in mixed infection
Source: PLoS Pathog. 2024 Jan 12;20(1):e1011941. doi: 10.1371/journal.ppat.1011941 (PMC10810479; doi:10.1371/journal.ppat.1011941)
Supplement: S5 Dataset — The start and stop codons of viral ORFs are coloured in red and underlined, the CAAT and TATA-boxes of the promoters coloured in brick red, the TATA-associated composite element (TACE) and conserved late elements (CLE) highlighted in green and cyan, respectively, the iterons highlighted in grey and SNPs and indels highlighted in yellow. (PDF) [file ppat.1011941.s010.pdf]

S5 Dataset. Reference sequences of the viral genome IL and IS76 and their pairwise alignment. The start and stop codons of viral ORFs are coloured in red and underlined, the CAAT and TATA-boxes of the promoters coloured in brick red, the TATA-associated composite element (TACE) and conserved late elements (CLE) highlighted in green and cyan, respectively, the iterons highlighted in grey and SNPs and indels highlighted in yellow.

```
>AM409201.1 Tomato Yellow Leaf Curl Virus complete genome, strain IL
ACCGGATGGCCGCGCCTTTTCCTTTTATGTGGTCCCCACGAGGGTTCCACAGACGTCACTGTCAACCAATCAAATTGCATACTCAAACGTTAGATAAGTGTTCATTTGTCTTTATATACTTGGTCCCCAAGTATTTTGTCTTGC
AATATGTGGGACCCACTTCTAAATGAATTTCCTGAATCTGTTCACGGATTTCGTTGTATGTTAGCTATTAAATATTTGCAGTCCGTTGAGGAAACTTACGAGCCCAATACATTGGGCCACGATTTAATTAGGGATCTTATATCT
GTTGTAAGGGCCCGTGACTATGTCGAAGCGACCAGGCGATATAATCATTTCCACGCCCGTTTCGAAGGTTCGCCGAAGGCTGAACTTCGACAGCCCATACAGCAGCCGTGCTGCTGTCCCCATTGTCCAAGGCACAAACAAGCG
ACGATCATGGACGTACAGGCCCATGTACCGAAAGCCCAGAATATACAGAATGTATCGAAGCCCTGATGTTCCCCGTGGATGTGAAGGCCCATGTAAAGTCCAGTCTTATGAGCAACGGGATGATATTAAGCACACTGGTATTGT
TCGTTGTGTTAGTGATGTTACTCGTGGATCTGGAATTACTCACAGAGTGGGTAAGAGGTTCTGTGTTAAATCGATATATTTTTTAGGTAAAGTCTGGATGGATGAAAATATCAAGAAGCAGAATCACACTAATCAGGTCATGTT
CTTTTTGGTCCGTGATAGAAGGCCCTATGGAAGCAGCCCAATGGATTTTGGACAGGTTTTTAATATGTTCGATAATGAGCCCAGTACCGCAACCGTGAAGAATGATTTGCGTGATAGGTTTCAAGTGATGAGAAAATTTCATGC
AACAGTTATTGGTGGGCCCTCTGGAATGAAGGAACAGGCATTAGTTAAGAGATTTTTTAAAATTAACAGTCATGTAACTTATAATCATCAGGAGGCAGCCAAGTACGAGAACCATACTGAAAACGCCTTATTATTGTATATGGC
ATGTACGCATGCCTCTAATCCAGTGTATGCAACTATGAAAATACGCATCTATTTCTATGATTCAATATCAAATTAATAAAATTTATATTTTATATCATGAGTTTCTGTTACATTTATTGTGTTTTCAAGTACATCATACAATAC
ATGATCAACTGCTCTGATTACATTGTTAATGGAAATTACACCAAGACTATCTACATACTTAAGAACTTCATATCTAAATACTCTTAAGAAATGACCAGTCTGAGGCTGTAATGTCGTCCAAATTCGGAAGTTGAGAAAACATTT
GTGAATCCCCATTACCTTCCTGATGTTGTGGTTGAATCTTATCTGAATGGAAATGATGTCGTGGTTCATTAGAAATGGCCGCTGGCTGTGTTCTGTTATCTTGAAATAGAGGGGATTGTTTATCTCCCAAATAAAAACGCCATT
CTCTGCCTGAGGAGCAGTGATGAGTTCCCCTGTGCGTGAATCCATGATTGTTGCAGTTGAGGTGGAGGTAGTATGAGCAGCCACAGTCTAGGTCTACACGCTTACGCCTTATTGGTTTCTTCTTGGCTATCTTGTGTTGGACCT
TGATTGATACTTGCGAACAGTGGCTCGTAGAGGGTGACGAAGGTTGCATTCTTGAGCGCCCAATTTTTCAAGGATATATTTTTTTCTTCGTCTAGATATTCCCTATATGAGGAGGTAGGTCCTGGATTGCAGAGGAAGATAGTG
GGAATTCCCCCTTTAATTTGAATGGGCTTCCCGTACTTTGTGTTGCTTTGCCAGTCCCTCTGGGCCCCCATGAATTCCTTGAAGTGCTTTAAATAATGCGGGTCTACGTCATCAATGACGTTGTACCACGCATTATTACTGTAC
ACCTTTGGGCTTAGGTCTAGATGTCCACATAAATAATTATGTGGGCCTAGAGACCTGGCCCACATTGTTTTGCCTGTTCTGCTATCACCCTCAATTACAATACTTATGGGTCTCCATGGCCGCGCAGCGGAAGACACGACGTTC
TCAGCGACCCACTCTTCAAGTTCATCTGGAACTTGATTAAAAGAAGAAGAAAGAAATGGAGAAACATAAACTTCTAAAGGAGGACTAAAAATCCTATCTAAATTTGAACTTAAATTATGAAATTGTAAAATATAGTCCTTTGGG
GCCTTCTCTTTTAATATATTGAGGGCCTCGGATTTATTGCCTGAATTGAGTGCTTCGGCATATGCGTCGTTGGCAGATTGCTGACCTCCTCTAGCTGATCTGCCATCGATTTGGAAAACTCCAAAATCAATGAAGTCTCCGTCT
TTCTCCACGTAGGTCTTGACATCTGTTGAGCTCTTAGCTGCCTGAATGTTCGGATGGAAATGTGCTGATCTGTTTGGGGATACCAGGTCGAAGAACCGTTGGTTCTTACATTGGTATTTGCCTTCGAATTGGATAAGCACATGG
AGATGTGGTTCCCCATTCTCGTGGAGTTCTCTGCAAACTTTGATGTATTTTTTATTTGTTGGGGTTTCTAGGTTTTTTAATTGGGAAAGTGCTTCCTCTTTAGAGAGAGAACAATTGGGATATGTTAGGAAATAATTTTTGGCA
TATATTTTAAATAAACGAGGCATGTTGAAATGAATCGGTGTCCCTCAAAGCTCTATGGCAATCGGTGTATCGGTGTCTTATTTATACCTGGACACCTAATGGCTATTTGGTAATTTCGTAAAAGTACATTGCAATTCAAAATTT
AAAATTCAAAAATCTAATCATTAAAGCGGCCATCCGTATAATATT
```

```
>LN812978.1 Tomato yellow leaf curl virus-Il complete genome, recombinant IS76
ACCGGATGGCCGCGCTCCCCGATAAAGTAGTAGGCCCTACGCAGTAATTTTTGTCGACCAATGAAAATGCAGCCTCAAACGTTAGATAAGTGTTCATTTGTCTTTATATACTTGGTCCCCAAGTAGTTTGTCTTGCAATATGTG
GGACCCACTTCTAAATGAATTTCCTGAATCTGTTCACGGATTTCGTTGTATGTTAGCTATTAAATATTTGCAGTCCGTTGAGGAAACTTACGAGCCCAATACATTGGGCCACGATTTAATTAGGGATCTTATATCTGTTGTAAG
GGCCCGTGACTATGTCGAAGCGGCCCGGCGATATAATCATTTCCACGCCCGCCTCGAAGGTTCGCCGAAGGCTGAACTTCGACAGCCCATACAACAGCCGTGCTGCTGTCCCCATTGTCCAAGGCACAAACAAGCGACGATCAT
GGACGTACAGGCCCATGTACCGAAAGCCCAGAATATACAGAATGTATCGAAGCCCTGATGTTCCCCGTGGATGTGAAGGCCCATGTAAAGTCCAGTCTTATGAGCAACGGGATGATATTAAGCACACTGGTATTGTTCGTTGTG
TTAGTGATGTTACTCGTGGATCTGGAATTACTCACAGAGTGGGTAAGAGGTTCTGTGTTAAATCGATATATTTTTTAGGTAAAGTCTGGATGGATGAAAATATCAAGAAGCAGAACCATACTAATCAGGTCATGTTCTTCTTGG
TCCGTGATAGAAGGCCCTATGGAAACAGCCCAATGGATTTTGGACAGGTTTTTAATATGTTCGATAATGAGCCCAGTACCGCAACAGTGAAGAATGATTTGCGGGATAGGTTTCAAGTGATGAGGAAATTTCATGCTACAGTTA
TTGGTGGACCCTCTGGAATGAAGGAACAGGCATTAGTTAAGAGGTTTTTTAGAATTAACAGTCATGTAACTTATAATCATCAGGAGGCAGCCAAGTACGAGAACCATACTGAAAACGCCTTGTTATTGTATATGGCATGTACGC
ATGCCTCTAATCCAGTGTATGCAACTATGAAAATACGCATCTATTTCTATGATTCAATATCAAATTAATAAAATTTGTATTTTATATCATGAGTTTCTGTTACATTTATTGTGTTTTCAAGTACATCATACAATACATGATCAA
CTGCTCTGATTACATTGTTAATGGAAATTACACCAAGACTATCTAAATACTTAAGAACTTCATATCTAAATACTCTTAAGAAATGACCAGTCTGAGGCTGTAATGTCGTCCAAATTCGGAAGTTGAGAAAACATTTGTGAATCC
CCATTACCTTCCTGATGTTGTGGTTGAATCTTATCTGAATGGAAATGATGTCGTTGTTCATTAGAAATGGCCGCTGGCTGTGTTCTGTTATCTTGAAATAGAGGGGATTGTTTATCTCCCAAATAAAAACGCCATTCTCTGCCT
GAGGAGCAGTGATGAGTTCCCCTGTGCGTGAATCCATGATTGTTGCAGTTGAGGTGGAGGTAGTATGAGCAGCCACAGTCTAGGTCTACACGCTTACGCCTTATTGGTTTCTTCTTGGCTATCTTGTGTTGGACCTTGATTGAT
ACTTGCGAACAGTGGCTCGTAGAGGGTGACGAAGGTTGCATTCTTGAGCGCCCAATTTTTCAAGGATATATTTTTTTCTTCGTCTAGATATTCCCTATATGAGGAGGTAGGTCCTGGATTGCAGAGGAAGATAGTGGGAATTCC
CCCTTTAATTTGAATGGGCTTCCCGTACTTTGTGTTGCTTTGCCAGTCCCTCTGGGCCCCCATGAATTCCTTGAAGTGCTTTAAATAATGCGGGTCTACGTCATCAATGACGTTGTACCACGCATCATTACTGTACACCTTTGG
GCTTAGGTCTAGATGTCCACATAAATAATTATGTGGGCCTAGAGACCTGGCCCACATTGTTTTGCCTGATCTGCTATCACCCTCAATTACAATACTCATGGGTCTCCATGGCCGCGCAGCGGAAGACACGACGTTCTCAGCGAC
CCACTCTTCAAGTTCATCTGGAACTTGATTAAAAGAAGAAGAAAGAAATGGAGAAACATAAACTTCTAAAGGAGGACTAAAAATCCTATCTAAATTTGAACTTAAATTATGAAATTGTAAAATATAGTCCTTTGGGGCCTTCTC
TTTTAATATATTGAGGGCCTCGGATTTATTGCCTGAATTGAGTGCTTCGGCATATGCGTCGTTGGCAGATTGCTGACCTCCTCTAGCTGATCTGCCATCGATTTGGAAAACTCCAAAATCAATGAAGTCTCCGTCTTTCTCCAC
GTAGGTCTTGACATCTGTTGAGCTCTTAACTGCCTGAATGTTCGGATGGAAATGTGCTGATCTGTTTGGGGATACCAGGTCGAAGAACCGTTGGTTCTTACATTGGTATTTGCCTTCGAATTGGATAAGCACATGGAGATGTGG
TTCCCCATTCTCGTGGAGTTCTCTGCAAACTTTGATGTATTTTTTATTTGTTGGGGTTTCTAGGTTTTTTAATTGGGAAAGTGCTTCCTCTTTAGAGAGAGAACAATTGGGATATGTTAGGAAATAATTTTTGGCATATATTTT
AAATAAACGAGGCATGTTGAAATGAATCGGTGTCCCTCAAAGCTCTATGGCAATCGGTGTATCGGTGTCTTATTTATACCTGGACACCTAATGGCTATTTGGTAATTTCGTAAAAGTACATTGCAATTCAAAATTCAAAATTCA
AAAATCAAATCATTAAAGCGGCCATCCGTATAATATT
```

```
LN812978_IS76      1 ACCGGATGGCCGCGC--TCCCCGATAAAGTAGTAGGCCCTACG-------     41
                     |||||||||||||||  |..||..|.|.||.||   |||.|||
AM409201_IL        1 ACCGGATGGCCGCGCCTTTTCCTTTTATGTGGT---CCCCACGAGGGTTC     47 CLE (conserved late element)

LN812978_IS76     42 --CAGTAATTTTTGTCGACCAATGAAAATGCAGCCTCAAACGTTAGATAA     89 CCAAT-box
                       |||...|...||||.||||||.|||.||||..||||||||||||||||
AM409201_IL       48 CACAGACGTCACTGTCAACCAATCAAATTGCATACTCAAACGTTAGATAA     97

LN812978_IS76     90 GTGTTCATTTGTCTTTATATACTTGGTCCCCAAGTAGTTTGTCTTGCAAT    139 TATA-box and transcription start site of the V2-V1 unit
                     ||||||||||||||||||||||||||||||||||||.|||||||||||||
AM409201_IL       98 GTGTTCATTTGTCTTTATATACTTGGTCCCCAAGTATTTTGTCTTGCAAT    147 TACE (TATA-associated composite element) with CLE

LN812978_IS76    140 ATGTGGGACCCACTTCTAAATGAATTTCCTGAATCTGTTCACGGATTTCG    189 Start codon of ORF V2 ORF
                     ||||||||||||||||||||||||||||||||||||||||||||||||||
AM409201_IL      148 ATGTGGGACCCACTTCTAAATGAATTTCCTGAATCTGTTCACGGATTTCG    197

LN812978_IS76    190 TTGTATGTTAGCTATTAAATATTTGCAGTCCGTTGAGGAAACTTACGAGC    239
                     ||||||||||||||||||||||||||||||||||||||||||||||||||
AM409201_IL      198 TTGTATGTTAGCTATTAAATATTTGCAGTCCGTTGAGGAAACTTACGAGC    247

LN812978_IS76    240 CCAATACATTGGGCCACGATTTAATTAGGGATCTTATATCTGTTGTAAGG    289
                     ||||||||||||||||||||||||||||||||||||||||||||||||||
AM409201_IL      248 CCAATACATTGGGCCACGATTTAATTAGGGATCTTATATCTGTTGTAAGG    297

LN812978_IS76    290 GCCCGTGACTATGTCGAAGCGGCCCGGCGATATAATCATTTCCACGCCCG    339
                     |||||||||||||||||||||.||.|||||||||||||||||||||||||
AM409201_IL      298 GCCCGTGACTATGTCGAAGCGACCAGGCGATATAATCATTTCCACGCCCG    347

LN812978_IS76    340 CCTCGAAGGTTCGCCGAAGGCTGAACTTCGACAGCCCATACAACAGCCGT    389
                     ..||||||||||||||||||||||||||||||||||||||||.|||||||
AM409201_IL      348 TTTCGAAGGTTCGCCGAAGGCTGAACTTCGACAGCCCATACAGCAGCCGT    397

LN812978_IS76    390 GCTGCTGTCCCCATTGTCCAAGGCACAAACAAGCGACGATCATGGACGTA    439
                     ||||||||||||||||||||||||||||||||||||||||||||||||||
AM409201_IL      398 GCTGCTGTCCCCATTGTCCAAGGCACAAACAAGCGACGATCATGGACGTA    447

LN812978_IS76    440 CAGGCCCATGTACCGAAAGCCCAGAATATACAGAATGTATCGAAGCCCTG    489
                     ||||||||||||||||||||||||||||||||||||||||||||||||||
AM409201_IL      448 CAGGCCCATGTACCGAAAGCCCAGAATATACAGAATGTATCGAAGCCCTG    497

LN812978_IS76    490 ATGTTCCCCGTGGATGTGAAGGCCCATGTAAAGTCCAGTCTTATGAGCAA    539
                     ||||||||||||||||||||||||||||||||||||||||||||||||||
AM409201_IL      498 ATGTTCCCCGTGGATGTGAAGGCCCATGTAAAGTCCAGTCTTATGAGCAA    547

LN812978_IS76    540 CGGGATGATATTAAGCACACTGGTATTGTTCGTTGTGTTAGTGATGTTAC    589
                     ||||||||||||||||||||||||||||||||||||||||||||||||||
AM409201_IL      548 CGGGATGATATTAAGCACACTGGTATTGTTCGTTGTGTTAGTGATGTTAC    597

LN812978_IS76    590 TCGTGGATCTGGAATTACTCACAGAGTGGGTAAGAGGTTCTGTGTTAAAT    639
                     ||||||||||||||||||||||||||||||||||||||||||||||||||
AM409201_IL      598 TCGTGGATCTGGAATTACTCACAGAGTGGGTAAGAGGTTCTGTGTTAAAT    647

LN812978_IS76    640 CGATATATTTTTTAGGTAAAGTCTGGATGGATGAAAATATCAAGAAGCAG    689
                     ||||||||||||||||||||||||||||||||||||||||||||||||||
AM409201_IL      648 CGATATATTTTTTAGGTAAAGTCTGGATGGATGAAAATATCAAGAAGCAG    697

LN812978_IS76    690 AACCATACTAATCAGGTCATGTTCTTCTTGGTCCGTGATAGAAGGCCCTA    739
                     ||.||.||||||||||||||||||||.|||||||||||||||||||||||
AM409201_IL      698 AATCACACTAATCAGGTCATGTTCTTTTTGGTCCGTGATAGAAGGCCCTA    747
```

```
LN812978_IS76    740 TGGAAACAGCCCAATGGATTTTGGACAGGTTTTTAATATGTTCGATAATG    789
                     |||||.||||||||||||||||||||||||||||||||||||||||||||
AM409201_IL      748 TGGAAGCAGCCCAATGGATTTTGGACAGGTTTTTAATATGTTCGATAATG    797

LN812978_IS76    790 AGCCCAGTACCGCAACAGTGAAGAATGATTTGCGGGATAGGTTTCAAGTG    839
                     ||||||||||||||||.|||||||||||||||||.|||||||||||||||
AM409201_IL      798 AGCCCAGTACCGCAACCGTGAAGAATGATTTGCGTGATAGGTTTCAAGTG    847

LN812978_IS76    840 ATGAGGAAATTTCATGCTACAGTTATTGGTGGACCCTCTGGAATGAAGGA    889
                     |||||.|||||||||||.||||||||||||||.|||||||||||||||||
AM409201_IL      848 ATGAGAAAATTTCATGCAACAGTTATTGGTGGGCCCTCTGGAATGAAGGA    897

LN812978_IS76    890 ACAGGCATTAGTTAAGAGGTTTTTAGAATTAACAGTCATGTAACTTATA     939
                     ||||||||||||||||||.||||||.||||||||||||||||||||||||
AM409201_IL      898 ACAGGCATTAGTTAAGAGATTTTTAAAATTAACAGTCATGTAACTTATA     947

LN812978_IS76    940 ATCATCAGGAGGCAGCCAAGTACGAGAACCATACTGAAAACGCCTTGTTA    989
                     ||||||||||||||||||||||||||||||||||||||||||||||.|||
AM409201_IL      948 ATCATCAGGAGGCAGCCAAGTACGAGAACCATACTGAAAACGCCTTATTA    997

LN812978_IS76    990 TTGTATATGGCATGTACGCATGCCTCTAATCCAGTGTATGCAACTATGAA   1039
                     ||||||||||||||||||||||||||||||||||||||||||||||||||
AM409201_IL      998 TTGTATATGGCATGTACGCATGCCTCTAATCCAGTGTATGCAACTATGAA   1047

LN812978_IS76   1040 AATACGCATCTATTTCTATGATTCAATATCAAATTAATAAAATTTGTATT   1089  Overlapping stop codons of C3 (TTA) and V1 (TAA) ORFs
                     |||||||||||||||||||||||||||||||||||||||||||||.||||        which define here borders of the leftward (C1-C4
AM409201_IL     1048 AATACGCATCTATTTCTATGATTCAATATCAAATTAATAAAATTTATATT   1097  and C2-C3) and the rightward (V2-V1) transcription units

LN812978_IS76   1090 TTATATCATGAGTTTCTGTTACATTTATTGTGTTTTCAAGTACATCATAC   1139
                     ||||||||||||||||||||||||||||||||||||||||||||||||||
AM409201_IL     1098 TTATATCATGAGTTTCTGTTACATTTATTGTGTTTTCAAGTACATCATAC   1147

LN812978_IS76   1140 AATACATGATCAACTGCTCTGATTACATTGTTAATGGAAATTACACCAAG   1189
                     ||||||||||||||||||||||||||||||||||||||||||||||||||
AM409201_IL     1148 AATACATGATCAACTGCTCTGATTACATTGTTAATGGAAATTACACCAAG   1197

LN812978_IS76   1190 ACTATCTAAATACTTAAGAACTTCATATCTAAATACTCTTAAGAAATGAC   1239
                     ||||||||.|||||||||||||||||||||||||||||||||||||||||
AM409201_IL     1198 ACTATCTACATACTTAAGAACTTCATATCTAAATACTCTTAAGAAATGAC   1247

LN812978_IS76   1240 CAGTCTGAGGCTGTAATGTCGTCCAAATTCGGAAGTTGAGAAAACATTTG   1289
                     ||||||||||||||||||||||||||||||||||||||||||||||||||
AM409201_IL     1248 CAGTCTGAGGCTGTAATGTCGTCCAAATTCGGAAGTTGAGAAAACATTTG   1297

LN812978_IS76   1290 TGAATCCCCATTACCTTCCTGATGTTGTGGTTGAATCTTATCTGAATGGA   1339
                     ||||||||||||||||||||||||||||||||||||||||||||||||||
AM409201_IL     1298 TGAATCCCCATTACCTTCCTGATGTTGTGGTTGAATCTTATCTGAATGGA   1347

LN812978_IS76   1340 AATGATGTCGTTGTTCATTAGAAATGGCCGCTGGCTGTGTTCTGTTATCT   1389
                     |||||||||||.||||||||||||||||||||||||||||||||||||||
AM409201_IL     1348 AATGATGTCGTGGTTCATTAGAAATGGCCGCTGGCTGTGTTCTGTTATCT   1397

LN812978_IS76   1390 TGAAATAGAGGGGATTGTTTATCTCCCAAATAAAAACGCCATTCTCTGCC   1439
                     ||||||||||||||||||||||||||||||||||||||||||||||||||
AM409201_IL     1398 TGAAATAGAGGGGATTGTTTATCTCCCAAATAAAAACGCCATTCTCTGCC   1447

LN812978_IS76   1440 TGAGGAGCAGTGATGAGTTCCCCTGTGCGTGAATCCATGATTGTTGCAGT   1489
                     ||||||||||||||||||||||||||||||||||||||||||||||||||
AM409201_IL     1448 TGAGGAGCAGTGATGAGTTCCCCTGTGCGTGAATCCATGATTGTTGCAGT   1497

LN812978_IS76   1490 TGAGGTGGAGGTAGTATGAGCAGCCACAGTCTAGGTCTACACGCTTACGC   1539
                     ||||||||||||||||||||||||||||||||||||||||||||||||||
AM409201_IL     1498 TGAGGTGGAGGTAGTATGAGCAGCCACAGTCTAGGTCTACACGCTTACGC   1547

LN812978_IS76   1540 CTTATTGGTTTCTTCTTGGCTATCTTGTGTTGGACCTTGATTGATACTTG   1589
                     ||||||||||||||||||||||||||||||||||||||||||||||||||
AM409201_IL     1548 CTTATTGGTTTCTTCTTGGCTATCTTGTGTTGGACCTTGATTGATACTTG   1597

LN812978_IS76   1590 CGAACAGTGGCTCGTAGAGGGTGACGAAGGTTGCATTCTTGAGCGCCCAA   1639  Start codon of C2 ORF
                     ||||||||||||||||||||||||||||||||||||||||||||||||||
AM409201_IL     1598 CGAACAGTGGCTCGTAGAGGGTGACGAAGGTTGCATTCTTGAGCGCCCAA   1647

LN812978_IS76   1640 TTTTTCAAGGATATATTTTTTTCTTCGTCTAGATATTCCCTATATGAGGA   1689 Transcription start site and TATA-box of the C2-C3 unit
                     ||||||||||||||||||||||||||||||||||||||||||||||||||
AM409201_IL     1648 TTTTTCAAGGATATATTTTTTTCTTCGTCTAGATATTCCCTATATGAGGA   1697

LN812978_IS76   1690 GGTAGGTCCTGGATTGCAGAGGAAGATAGTGGGAATTCCCCCTTTAATTT   1739
                     ||||||||||||||||||||||||||||||||||||||||||||||||||
AM409201_IL     1698 GGTAGGTCCTGGATTGCAGAGGAAGATAGTGGGAATTCCCCCTTTAATTT   1747

LN812978_IS76   1740 GAATGGGCTTCCCGTACTTTGTGTTGCTTTGCCAGTCCCTCTGGGCCCCC   1789
                     ||||||||||||||||||||||||||||||||||||||||||||||||||
AM409201_IL     1748 GAATGGGCTTCCCGTACTTTGTGTTGCTTTGCCAGTCCCTCTGGGCCCCC   1797

LN812978_IS76   1790 ATGAATTCCTTGAAGTGCTTTAAATAATGCGGGTCTACGTCATCAATGAC   1839
                     ||||||||||||||||||||||||||||||||||||||||||||||||||
AM409201_IL     1798 ATGAATTCCTTGAAGTGCTTTAAATAATGCGGGTCTACGTCATCAATGAC   1847

LN812978_IS76   1840 GTTGTACCACGCATCATTACTGTACACCTTTGGGCTTAGGTCTAGATGTC   1889
                     ||||||||||||||.|||||||||||||||||||||||||||||||||||
AM409201_IL     1848 GTTGTACCACGCATTATTACTGTACACCTTTGGGCTTAGGTCTAGATGTC   1897

LN812978_IS76   1890 CACATAAATAATTATGTGGGCCTAGAGACCTGGCCCACATTGTTTTGCCT   1939
                     ||||||||||||||||||||||||||||||||||||||||||||||||||
AM409201_IL     1898 CACATAAATAATTATGTGGGCCTAGAGACCTGGCCCACATTGTTTTGCCT   1947

LN812978_IS76   1940 GATCTGCTATCACCCTCAATTACAATACTCATGGGTCTCCATGGCCGCGC   1989
                     |.|||||||||||||||||||||||||||.||||||||||||||||||||
AM409201_IL     1948 GTTCTGCTATCACCCTCAATTACAATACTTATGGGTCTCCATGGCCGCGC   1997

LN812978_IS76   1990 AGCGGAAGACACGACGTTCTCAGCGACCCACTCTTCAAGTTCATCTGGAA   2039
                     ||||||||||||||||||||||||||||||||||||||||||||||||||
AM409201_IL     1998 AGCGGAAGACACGACGTTCTCAGCGACCCACTCTTCAAGTTCATCTGGAA   2047

LN812978_IS76   2040 CTTGATTAAAAGAAGAAGAAAGAAATGGAGAAACATAAACTTCTAAAGGA   2089
                     ||||||||||||||||||||||||||||||||||||||||||||||||||
AM409201_IL     2048 CTTGATTAAAAGAAGAAGAAAGAAATGGAGAAACATAAACTTCTAAAGGA   2097

LN812978_IS76   2090 GGACTAAAAATCCTATCTAAATTTGAACTTAAATTATGAAATTGTAAAAT   2139
                     ||||||||||||||||||||||||||||||||||||||||||||||||||
AM409201_IL     2098 GGACTAAAAATCCTATCTAAATTTGAACTTAAATTATGAAATTGTAAAAT   2147

LN812978_IS76   2140 ATAGTCCTTTGGGGCCTTCTCTTTTAATATATTGAGGGCCTCGGATTTAT   2189
                     ||||||||||||||||||||||||||||||||||||||||||||||||||
```

```
AM409201_IL     2148 ATAGTCCTTTGGGGCCTTCTCTTTTAATATATTGAGGGCCTCGGATTTAT   2197

LN812978_IS76   2190 TGCCTGAATTGAGTGCTTCGGCATATGCGTCGTTGGCAGATTGCTGACCT   2239
                     ||||||||||||||||||||||||||||||||||||||||||||||||||
AM409201_IL     2198 TGCCTGAATTGAGTGCTTCGGCATATGCGTCGTTGGCAGATTGCTGACCT   2247

LN812978_IS76   2240 CCTCTAGCTGATCTGCCATCGATTTGGAAAACTCCAAAATCAATGAAGTC   2289
                     ||||||||||||||||||||||||||||||||||||||||||||||||||
AM409201_IL     2248 CCTCTAGCTGATCTGCCATCGATTTGGAAAACTCCAAAATCAATGAAGTC   2297

LN812978_IS76   2290 TCCGTCTTTCTCCACGTAGGTCTTGACATCTGTTGAGCTCTTAACTGCCT   2339
                     |||||||||||||||||||||||||||||||||||||||||||.||||||
AM409201_IL     2298 TCCGTCTTTCTCCACGTAGGTCTTGACATCTGTTGAGCTCTTAGCTGCCT   2347

LN812978_IS76   2340 GAATGTTCGGATGGAAATGTGCTGATCTGTTTGGGGATACCAGGTCGAAG   2389
                     ||||||||||||||||||||||||||||||||||||||||||||||||||
AM409201_IL     2348 GAATGTTCGGATGGAAATGTGCTGATCTGTTTGGGGATACCAGGTCGAAG   2397

LN812978_IS76   2390 AACCGTTGGTTCTTACATTGGTATTTGCCTTCGAATTGGATAAGCACATG   2439
                     ||||||||||||||||||||||||||||||||||||||||||||||||||
AM409201_IL     2398 AACCGTTGGTTCTTACATTGGTATTTGCCTTCGAATTGGATAAGCACATG   2447

LN812978_IS76   2440 GAGATGTGGTTCCCCATTCTCGTGGAGTTCTCTGCAAACTTTGATGTATT   2489
                     ||||||||||||||||||||||||||||||||||||||||||||||||||
AM409201_IL     2448 GAGATGTGGTTCCCCATTCTCGTGGAGTTCTCTGCAAACTTTGATGTATT   2497

LN812978_IS76   2490 TTTTATTTGTTGGGGTTTCTAGGTTTTTTAATTGGGAAAGTGCTTCCTCT   2539
                     ||||||||||||||||||||||||||||||||||||||||||||||||||
AM409201_IL     2498 TTTTATTTGTTGGGGTTTCTAGGTTTTTTAATTGGGAAAGTGCTTCCTCT   2547

LN812978_IS76   2540 TTAGAGAGAGAACAATTGGGATATGTTAGGAAATAATTTTTGGCATATAT   2589
                     ||||||||||||||||||||||||||||||||||||||||||||||||||
AM409201_IL     2548 TTAGAGAGAGAACAATTGGGATATGTTAGGAAATAATTTTTGGCATATAT   2597

LN812978_IS76   2590 TTTAAATAAACGAGGCATGTTGAAATGAATCGGTGTCCCTCAAAGCTCTA   2639   C1 ORF start codon and transcription start site
                     ||||||||||||||||||||||||||||||||||||||||||||||||||
AM409201_IL     2598 TTTAAATAAACGAGGCATGTTGAAATGAATCGGTGTCCCTCAAAGCTCTA   2647   Iterons

LN812978_IS76   2640 TGGCAATCGGTGTATCGGTGTCTTATTTATACCTGGACACCTAATGGCTA   2689   TATA-box of the C1-C4 unit
                     ||||||||||||||||||||||||||||||||||||||||||||||||||
AM409201_IL     2648 TGGCAATCGGTGTATCGGTGTCTTATTTATACCTGGACACCTAATGGCTA   2697   Iterons

LN812978_IS76   2690 TTTGGTAATTTCGTAAAAGTACATTGCAATTCAAAATTCAAAATTCAAAA   2739
                     ||||||||||||||||||||||||||||||||||||||.|||||||||||
AM409201_IL     2698 TTTGGTAATTTCGTAAAAGTACATTGCAATTCAAAATTTAAAATTCAAAA   2747

LN812978_IS76   2740 ATCAAATCATTAAAGCGGCCATCCGTATAATATT   2773
                     |||.||||||||||||||||||||||||||||||
AM409201_IL     2748 ATCTAATCATTAAAGCGGCCATCCGTATAATATT   2781
```
